# Total Syntheses of Colletopeptide A and Colletotrichamide A

**DOI:** 10.3390/molecules28207194

**Published:** 2023-10-20

**Authors:** Jing Chen, Yangyang Jiang, Jialei Yan, Chao Xu, Tao Ye

**Affiliations:** 1 State Key Laboratory of Chemical Oncogenomics, Peking University Shenzhen Graduate School, Shenzhen 518055, China; chenjingchey@pku.edu.cn (J.C.); yangyangjiang@stu.pku.edu.cn (Y.J.); 2 Innovation Center of Marine Biotechnology and Pharmaceuticals, School of Biotechnology and Health Sciences, Wuyi University, Jiangmen 529020, China; yanjialei@wyu.edu.cn; 3 QianYan (Shenzhen) Pharmatech. Ltd., Building-3, Longcheng Industrial Park, Qinglin Road West, Longgang District, Shenzhen 518172, China

**Keywords:** total synthesis, natural products, asymmetric crotylation, glycosylation

## Abstract

The first total syntheses of cyclic depsipeptides colletopeptide A and colletotrichamide A, have been accomplished. The key advanced intermediate, a cyclic tridepsipeptide derivative, was constructed using a sequence of transformations that features asymmetric Brown crotylation, cross metathesis, Yamaguchi esterification, ozonolysis, and macrolactamization. A late-stage incorporation of the mannose fragment completed the synthesis of colletotrichamide A, and the desilylation of the common intermediate gave rise to colletopeptide A, which led to unambiguous confirmation of the absolute stereochemistry of the aforementioned natural products.

## 1. Introduction

Cyclic depsipeptides are a class of heterocyclic peptides that have ester bonds (depsi) in the core structure and are formed by substituting one or more amide bonds with lactone bonds in the peptide skeleton [1]. Cyclic depsipeptides were isolated from bacteria [2,3], fungi [4,5,6], as well as other marine organisms [7,8,9,10], and displayed a wide range of biological activities, such as cytotoxic [11], anticancer [12], antifungal [6,13], antibacterial [14], antimalarial [6,15], and insecticidal [16]. The macrocyclic framework of cyclic depsipeptides reduced the conformational flexibility, allowing deeper penetration into the membrane and thereby increasing the bioactivity [17]. Moreover, cyclic depsipeptides also exhibit a better resistance profile to proteases than their linear counterparts [18]. A large variety of bioactive cyclic depsipeptides have been isolated and characterized, which occupy therapeutically relevant chemical space that could lead to the discovery of new biology, chemical tools, or even drug leads [19,20,21,22,23,24].

**Figure 1:**
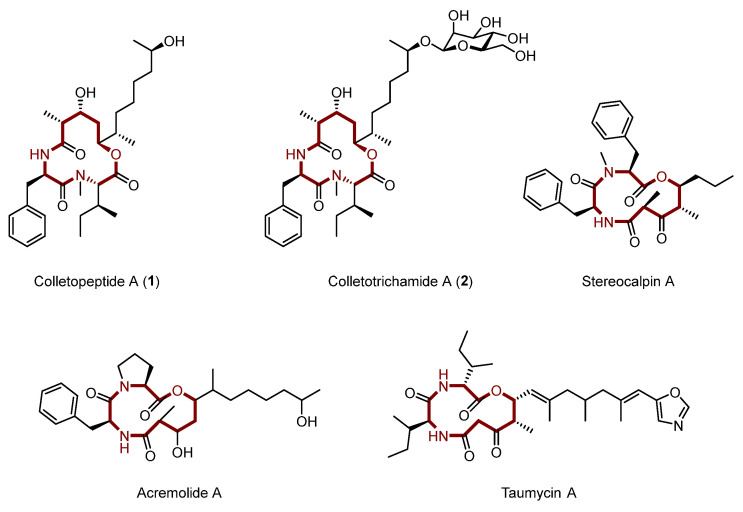
Tridepsipeptide natural products with 12-membered cyclic ring.

Colletopeptide A and colletotrichamide A are natural cyclic depsipeptides independently isolated by two groups in 2019 from the endophytic fungus *Colletotrichum* sp. S8 and *Colletotrichum gloeosporioides* JS419 (Figure 1) [25,26]. The structures of these two compounds were elucidated on the basis of extensive spectroscopic analysis. The absolute configuration of colletopeptide A was confirmed via X-ray crystallographic analysis, while the configuration of colletotrichamide A was assigned using the modified Mosher’s method, the advanced Marfey’s method, and sugar derivatization. Colletopeptide A inhibited lipopolysaccharide-induced nitric oxide production in RAW264.7 macrophages with the IC_50_ values of 8.3 μM, which suppressed the production of inflammatory factors IL-6 and TNF-α and decreased the phosphorylation of NF-κB-associated proteins IκBα and p65. Our group has been interested for some time in the synthesis of cyclic depsipeptides [27,28,29,30,31,32,33] and believed that the further studies of these natural products might provide a potential lead compound for drug discovery and development efforts. In the context of this research program, we decided to embark on studies directed toward the total synthesis of colletopeptide A and colletotrichamide A. Herein, we provide the details of our synthetic studies.

**Scheme 1:**
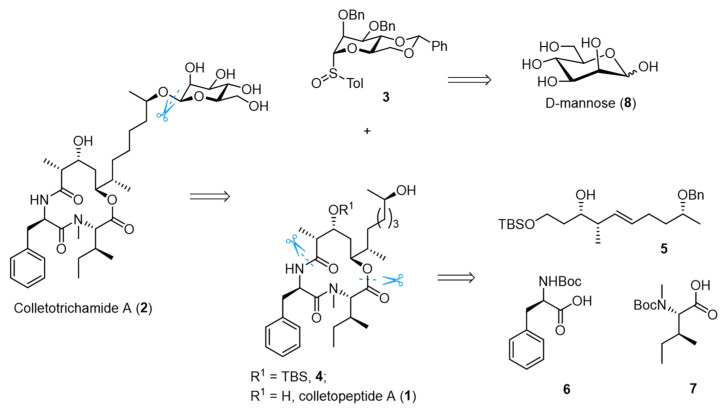
Retrosynthetic analysis of colletopepetide A (**1**) and colletotrichamide A (**2**).

Our strategy for the synthesis of colletopepetide A (**1**) and colletotrichamide A (**2**) is outlined in Scheme 1. It is envisaged that a late-stage glycosylation of the aglycone fragment **4** with the d-mannose-derived thioglycosyl donor **3** would produce the natural product in its protected form. Careful inspection of the complete aglycone framework reveals that the 12-membered macrocycle could be assembled from subunit **5** and Boc-d-Phe **6**, Boc-*N*-Me-l-Ile **7**.

## 2. Results and Discussion

**Scheme 2:**
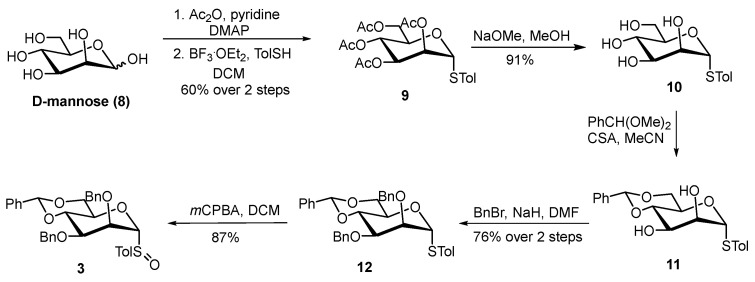
Synthesis of glycosyl donor **3**.

Our synthesis of colletotrichamide A (**2**) commenced from the commercially available D-mannose (**8**), which was converted into the thiomannoside **9** via pentaacylation of the mannose followed by the Lewis acid catalyzed displacement of the anomeric acetate group with *p*-thiocresol (Scheme 2) [34]. The removal of the remaining acetate groups under the basic condition gave tetra-ol **10**, which was condensed with benzaldehyde dimethyl acetal in the presence of a catalytic amount of CSA to give the six-membered cyclic acetal **11** exclusively. Further protection of the remaining two hydroxyl groups as benzyl ether and the subsequent oxidation with *m*-CPBA delivered the corresponding glycosyl donor **3** (66% yield over three steps).

**Scheme 3:**
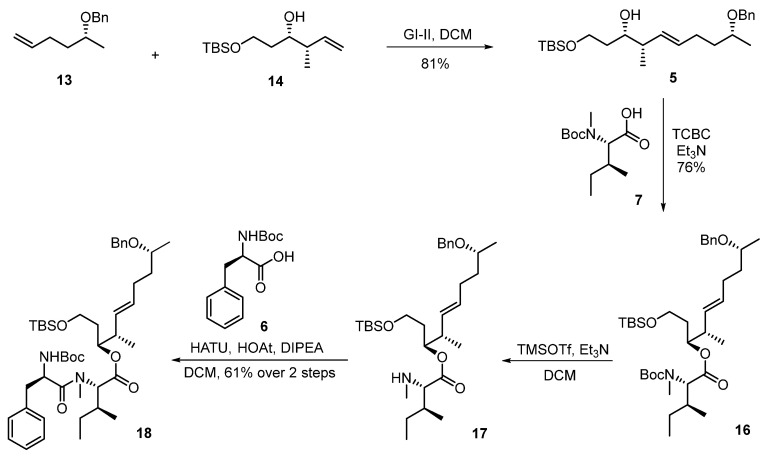
Synthesis of linear tridepsipeptide **18**.

With glycosyl donor **3** in hand, we turned our attention to the synthesis of the depsipeptide core. Thus, cross metathesis between the known alkenes **13** [35] and **14** [36] in the presence of the Grubbs II catalyst afforded alkene **5** in an 81% yield (Scheme 3). The subsequent Yamaguchi esterification of alcohol **5** with acid **7** led to the formation of ester **16**, followed by treatment with trimethylsilyl trifluromethanesulfonate using the Ohfune procedure [37], which provided amino ester **17** and which underwent an amide coupling reaction with Boc-d-Phe **6** to give rise to the linear tridepsipeptide **18** in a 61% yield over two steps.

**Scheme 4:**
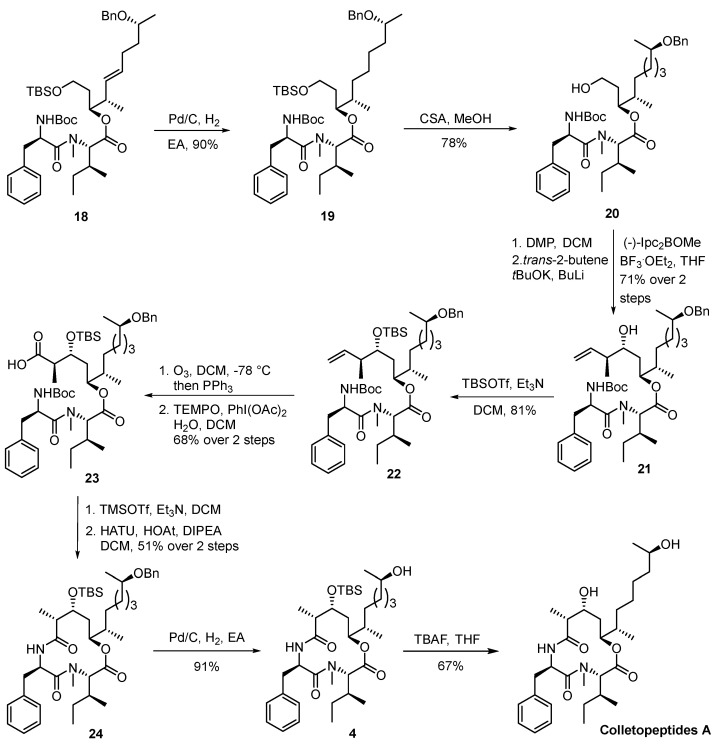
Synthesis of aglycone fragment **4** and colletopepetide A (**1**).

The stage was now set for converting the linear tridepsipeptide **18** to aglycone fragment **4** (Scheme 4). The exposure of the tridepsipeptide **18** directly to H_2_ in the presence of 5% Pd/C led to selective hydrogenation of the olefin exclusively, without causing the hydrogenolysis of the benzyl ether. The removal of the TBS-protecting group in **19** delivered the primary alcohol intermediate **20** in a 78% yield. The treatment of alcohol **20** with the Dess–Martin reagent provided the corresponding aldehyde, which was immediately subjected to Brown asymmetric crotylation conditions using *trans*-2-butene and (−)-diisopinylcampheylmethoxyborane to afford the corresponding homoallylic alcohol **21** in a 71% yield [38]. The resulting homoallylic alcohol **21** was protected as its *tert*-butyldimethylsilyl ether (**22**) before ozonolysis of the terminal alkene gave rise to the corresponding aldehyde and the subsequent oxidation utilizing TEMPO and PhI(OAc)_2_ provided acid **23** in a 68% yield over two steps. Next, the removal of the Boc carbamate of **23** could be accomplished using triethylsilyl trifluromethanesulfonate and triethylamine to deliver the ring-closing precursor which underwent macrolactamization via the action of HATU, HOAt, and *i*-Pr_2_NEt furnishing **24** in a 51% yield over two steps. The benzyl group on the side chain was removed via hydrogenolysis to provide the aglycone fragment **4**, and the final deprotection of the TBS group led to the completion of the natural product colletopepetide A (**1**) (3.2% overall yield).

**Scheme 5:**
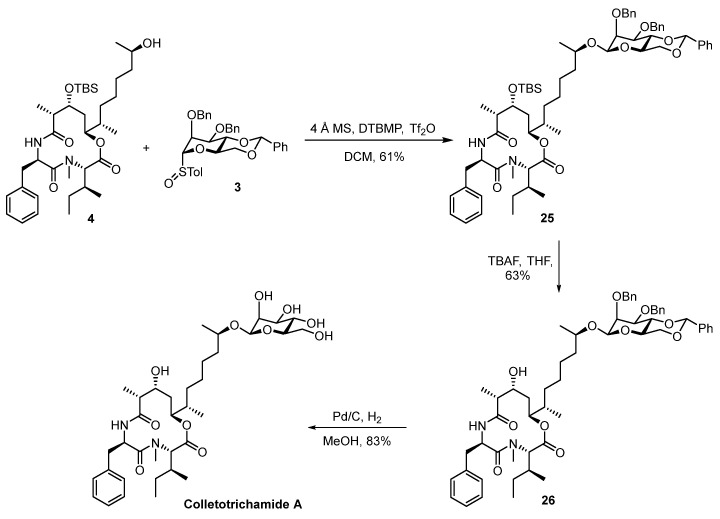
Completion of the synthesis of colletotrichamide A (**2**).

Next, we shifted our focus to the total synthesis of colletotrichamide A (**2**) (Scheme 5). As mentioned in the Introduction, the β-mannoside linkage in colletotrichamide A (**2**) was planned to be constructed via a sulfoxide-based glycosylation of the aglycone fragment **4** with the d-mannose-derived thioglycosyl donor **3** [39,40]. This process is based on the conventional activation of thioglycosyl sulfoxides with in situ triflic anhydride-generated sulfenyl triflates as powerful promoters in a glycosidic-bond-forming reaction. In the event, this key glycosylation reaction proceeded smoothly, providing glycosylated product **25** as the sole detectable stereoisomer in a 61% yield. Sequential deprotections of both the benzyl and TBS groups of **25** under the previous conditions provided colletotrichamide A (**2**) in a 52% yield over two steps (1.5% overall yield). The spectroscopic data of colletopepetide A (**1**) (^1^H NMR and ^13^C NMR) and the high-resolution mass spectrometric data of the synthetic sample were in all respect identical to those recorded in the literature for the natural colletopepetide A. In addition, a negative optical rotation was noted, ${\left[\alpha \right]}_{D}^{20}$= −62.0 (*c* 0.8, MeOH), in close accord with the previously reported value ${\left[\alpha \right]}_{D}^{20}$ = −68.9 (*c* 0.044, MeOH), thereby providing further confirmation of the absolute stereochemistry of the natural colletopepetide A. The spectroscopic data of the synthetic colletotrichamide A (**2**) (^1^H NMR, ^13^C NMR, and HRMS) were fully consistent with the reported values. Since the successful total synthesis of colletopepetide A also confirmed the absolute stereochemistry of aglycone fragment **4**, the current synthesis of colletotrichamide A (**2**) from aglycone fragment **4** also confirms the structure and absolute configuration of colletotrichamide A. The specific optical rotation, ${\left[\alpha \right]}_{D}^{25}$ = −78.1 (*c* 1.0, MeOH) of the synthetic colletotrichamide A (**2**) closely matched the reported value ${\left[\alpha \right]}_{D}^{25}$ = −70.4 (*c* 0.0625, MeOH) [41] and established that the absolute configuration of natural colletotrichamide A is correctly represented in Scheme 5.

## 3. Materials and Methods

### 3.1. General

All reactions were conducted in flame-dried or oven-dried glassware under an atmosphere of dry nitrogen or argon. Oxygen and/or moisture-sensitive solids and liquids were transferred appropriately. The concentration of solutions in vacuo was accomplished using a rotary evaporator fitted with a water aspirator. Residual solvents were removed under a high vacuum (0.1–0.2 mm Hg). All reaction solvents were purified before use: tetrahydrofuran (THF) was distilled from Na/benzophenone. Toluene was distilled over molten sodium metal. Dichloromethane (DCM), 1,2-dichloroethane (DCE) and trimethylamine (Et_3_N) were distilled from CaH_2_. Methanol (MeOH) was distilled from Mg/I_2_. The reagents were purchased at the highest commercial quality and used without further purification unless otherwise stated. Flash column chromatography was performed using the indicated solvents on silica gel 60 (230–400 mesh ASTM E. Qingdao, Tsingtao, China). Reactions were monitored using thin-layer chromatography (TLC), which was carried out using pre-coated sheets (Qingdao silica gel 60-F250, 0.2 mm). Compounds were visualized with UV light, iodine, and ceric ammonium molybdate stainer phosphomolybdic acid in EtOH. The ^1^H NMR spectra were recorded using Avance 400 MHz, Avance 500 MHz, or Avance 600 MHz spectrometers (Bruker, Karlsruhe, Germany). Chemical shifts were reported in parts per million (ppm), relative to either a tetramethylsilane (TMS) internal standard or the signals due to the solvent. The following abbreviations are used to describe the spin multiplicity: s = singlet, d = doublet, t = triplet, q = quartet, qn = quintet, m = multiplet, br = broad, dd = doublet of doublets, dt = doublet of triplets, dq = doublet of quartets, ddd = doublet of doublet of doublets; other combinations are derived from those listed above. Coupling constants (J) are reported in Hertz (Hz) for the corresponding solutions, and chemical shifts are reported as parts per million (ppm) relative to residual CHCl_3_ δH (7.26 ppm). The ^13^C NMR nuclear magnetic resonance spectra were recorded at 100 MHz, 125 MHz, or 150 MHz for the corresponding solutions, and chemical shifts are reported as parts per million (ppm) relative to residual CDCl_3_ δC (77.16 ppm). The ^1^H and ^13^C NMR spectra of compounds **1**–**5**, **9**, **10**, **12**, **16**, and **18**–**26** are available in the Supplementary Materials (Figures S1–S36). The high-resolution mass spectra were measured on an ABI Q-star Elite (Beijing, China). Optical rotations were recorded on a Rudolph AutoPol-I polarimeter (Shanghai, China) at 589 nm with a 50 mm cell. The data are reported as follows: specific rotation (*c* (g/100 mL), solvent).

### 3.2. General Experimental Procedures

#### 3.2.1. (2*R*,3*R*,4*S*,5*S*,6*R*)-2-(acetoxymethyl)-6-(p-tolylthio)tetrahydro-2H-pyran-3,4,5-triyl triacetate (**9**)

To a solution of d-mannose (**8**) (5.0 g, 27.8 mmol, 1.0 equiv.) and Ac_2_O (19.7 mL, 208.5 mmol, 7.5 equiv.) in pyridine (80 mL, 0.35 M) at room temperature, DMAP (340 mg, 2.78 mmol, 0.1 equiv.) was added. The reaction mixture was stirred at room temperature for 13 h and then concentrated in vacuo, and the oily residue was redissolved in EtOAc (250 mL) and quenched with an HCl aqueous solution (50 mL, 1 N in H_2_O). The aqueous layer was extracted with EtOAc (3 × 30 mL) and the combined organic layers were washed with a saturated aqueous solution of NaHCO_3_ (50 mL) and brine (50 mL), dried over anhydrous Na_2_SO_4_, and then filtered and concentrated in vacuo. The residue was used directly in the next step without further purification.

To a solution of above crude acetate and TolSH (5.2 g, 41.7 mmol, 1.5 equiv.) in DCM (200 mL, 0.14 M) at 0 °C, BF_3_•OEt_2_ (10.3 mL, 83.4 mmol, 3.0 equiv.) was added. The mixture was stirred for 9 h at room temperature and then quenched with a saturated aqueous solution of NaHCO_3_ (50 mL). The aqueous layer was extracted with ethyl acetate (3 × 100 mL). The combined organic layers were washed with H_2_O (50 mL) and brine (50 mL), dried over anhydrous Na_2_SO_4_, and then filtered and concentrated in vacuo. Purification of the crude product was performed via flash chromatography on silica (hexanes/EtOAc = 1/1) to afford **9** (7.6 g, 60%) as a colorless oil. **TLC**: Rf = 0.4 (hexanes/EtOAc = 2/1), UV & PMA stain. **^1^H NMR** (400 MHz, CDCl_3_) δ 7.54–7.29 (m, 2H), 7.18–6.97 (m, 2H), 5.46 (dd, *J* = 2.7, 1.6 Hz, 1H), 5.38 (d, *J* = 1.7 Hz, 1H), 5.32–5.10 (m, 2H), 4.77–4.45 (m, 1H), 4.26 (dd, J = 12.2, 6.0 Hz, 1H), 4.07 (dd, *J* = 12.3, 2.4 Hz, 1H), 2.29 (s, 3H), 2.11 (s, 3H), 2.04 (s, 3H), 2.02 (s, 3H), 1.98 (s, 3H). **^13^C NMR** (100 MHz, CDCl_3_) δ 170.5, 169.8, 169.7, 169.7, 138.4, 132.6, 129.9, 128.8, 86.0, 70.9, 69.4, 69.4, 66.4, 62.5, 21.1, 20.8, 20.7, 20.7, 20.6.

#### 3.2.2. (2*R*,3*S*,4*S*,5*S*,6*R*)-2-(hydroxymethyl)-6-(*p*-tolylthio)tetrahydro-2H-pyran-3,4,5-triol (**10**)

To a solution of acetate **9** (3.0 g, 6.6 mmol, 1.0 equiv.) in MeOH (50 mL, 0.13 M) at room temperature, NaOMe (357 mg, 6.6 mmol, 1.0 equiv.) was added. The reaction mixture was stirred at room temperature for 9 h and then quenched with a saturated aqueous solution of NH_4_Cl (20 mL). The aqueous layer was extracted with ethyl acetate (3 × 30 mL) and the combined organic layers were washed with H_2_O (30 mL) and brine (30 mL), dried over anhydrous Na_2_SO_4_, and then filtered and concentrated in vacuo. Purification of the crude product was performed via flash chromatography on silica (MeOH/DCM = 1/10) to afford **10** (1.7 g, 91%) as a white solid. **TLC**: Rf = 0.4 (MeOH/DCM = 1/10), UV & PMA stain. **^1^H NMR** (400 MHz, MeOD) δ 7.41 (d, *J* = 8.2 Hz, 2H), 7.13 (d, *J* = 7.9 Hz, 2H), 5.35 (d, *J* = 1.6 Hz, 1H), 4.10–4.04 (m, 1H), 4.03 (dd, *J* = 5.6, 2.9 Hz, 1H), 3.86–3.73 (m, 2H), 3.75–3.63 (m, 2H), 2.31 (s, 3H). **^13^C NMR** (101 MHz, MeOD) δ 138.9, 133.5, 132.1, 130.7, 90.8, 75.5, 73.7, 73.1, 68.7, 62.6, 21.1.

#### 3.2.3. (2*R*,4a*R*,6*R*,7*S*,8*S*,8a*R*)-7,8-bis(benzyloxy)-2-phenyl-6-(*p*-tolylthio)hexahydropyrano [3,2-d][1,3]dioxine (**12**)

To a solution of tetra-ol **10** (1.0 g, 3.5 mmol, 1.0 equiv.) and benzaldehyde dimethyl acetal (0.56 mL, 3.7 mmol, 1.05 equiv.) in MeCN (50 mL, 0.07 M) at room temperature, camphorsulfonic acid (204 mg, 0.88 mmol, 0.25 equiv.) was added. The reaction mixture was heated to reflux under Argon and stirred for 3 h. After being cooled back to room temperature, the reaction mixture was quenched with a saturated aqueous solution of NaHCO_3_ (20 mL). The aqueous layer was extracted with ethyl acetate (3 × 30 mL) and the combined organic layers were washed with H_2_O (30 mL) and brine (30 mL), dried over anhydrous Na_2_SO_4_, and then filtered and concentrated in vacuo. The residue was used directly in the next step without further purification.

To a suspension of NaH (308 mg, 7.7 mmol, 60% dispersion in mineral oil, 2.2 equiv.) in DMF (50 mL, 0.07 M) at 0 °C, a solution of the above crude diol in DMF (10 mL) was added. A total of 30 min later, BnBr (0.92 mL, 7.7 mmol, 2.2 equiv.) was slowly added at 0 °C. The reaction mixture was warmed up to ambient temperature, stirred for 6 h, and then quenched with a saturated aqueous solution of NH_4_Cl (30 mL). The aqueous layer was extracted with ethyl acetate (3 × 50 mL) and the combined organic layers were washed with H_2_O (10 mL) and brine (10 mL), dried over anhydrous Na_2_SO_4_, and then filtered and concentrated in vacuo. Purification of the crude product was performed via flash chromatography on silica (hexanes/EtOAc = 6/1) to afford **12** (1.47 g, 76%) as a white solid. **TLC**: Rf = 0.5 (hexanes/EtOAc = 6/1), UV & PMA stain. **^1^H NMR** (400 MHz, CDCl_3_) δ 7.65–7.51 (m, 2H), 7.47–7.29 (m, 15H), 7.19–7.05 (m, 2H), 5.69 (s, 1H), 5.49 (t, *J* = 1.5 Hz, 1H), 4.87 (dd, *J* = 12.2, 2.0 Hz, 1H), 4.76 (d, *J* = 1.9 Hz, 2H), 4.70 (dd, *J* = 12.3, 2.0 Hz, 1H), 4.36 (qd, *J* = 6.3, 4.1 Hz, 2H), 4.27 (dd, *J* = 10.1, 3.6 Hz, 1H), 4.08 (dt, *J* = 2.9, 1.5 Hz, 1H), 4.03 (dt, *J* = 9.5, 3.0 Hz, 1H), 3.93 (td, *J* = 10.1, 8.8, 3.1 Hz, 1H), 2.37 (s, 3H). **^13^C NMR** (100 MHz, CDCl_3_) δ 138.5, 138.0, 137.9, 137.7, 132.4, 130.0, 130.0, 129.0, 128.5, 128.5, 128.3, 128.2, 127.9, 127.7, 127.7, 126.2, 101.6, 87.5, 79.2, 78.1, 76.3, 73.2, 73.1, 68.6, 65.5, 21.2. **HRMS (ESI)** calculated for C_34_H_35_O_5_S^+^ [M + H]^+^ 555.2205 found 555.2230.

#### 3.2.4. (2*R*,4a*R*,6*R*,7*S*,8*S*,8a*R*)-7,8-bis(benzyloxy)-2-phenyl-6-(*p*-tolylsulfinyl)hexahydropyrano[3,2-d][1,3]dioxine (**3**)

To a solution of thioether **12** (800 mg, 1.4 mmol, 1.0 equiv.) in DCM (30 mL, 0.05M) at –78 °C, *m*-CPBA (259 mg, 1.5 mmol, 1.1 equiv.) in DCM (5 mL) was added dropwise. The reaction mixture was warmed up to –35 °C, stirred for 2 h, and then quenched with a saturated aqueous solution of NaHCO_3_ (15 mL) and Na_2_S_2_O_3_ (15 mL). The aqueous layer was extracted with ethyl acetate (3 × 30 mL) and the combined organic layers were washed with H_2_O (20 mL) and brine (20 mL), dried over anhydrous Na_2_SO_4_, and then filtered and concentrated in vacuo. Purification of the crude product was performed via flash chromatography on silica (hexanes/EtOAc = 3/1) to afford **3** (695 mg, 87%) as a white solid. **TLC**: Rf = 0.4 (hexanes/EtOAc = 3/1), UV & PMA stain. **^1^H NMR** (400 MHz, CDCl_3_) δ 7.52–7.46 (m, 2H), 7.44–7.31 (m, 12H), 7.31–7.18 (m, 5H), 5.61 (s, 1H), 4.81 (d, *J* = 12.1 Hz, 1H), 4.66 (d, *J* = 12.1 Hz, 1H), 4.58 (s, 2H), 4.47 (d, *J* = 1.2 Hz, 1H), 4.40 (dd, *J* = 3.0, 1.4 Hz, 1H), 4.37–4.25 (m, 2H), 4.19 (dd, *J* = 10.2, 4.8 Hz, 1H), 4.09 (ddd, *J* = 13.2, 6.9, 3.1 Hz, 1H), 3.73 (t, *J* = 10.1 Hz, 1H), 2.41 (s, 3H). **^13^C NMR** (100 MHz, CDCl_3_) δ 142.2, 138.3, 138.2, 137.4, 137.3, 130.2, 129.0, 128.4, 128.3, 128.3, 128.2, 127.9, 127.8, 127.6, 126.1, 124.4, 101.6, 97.5, 78.1, 76.3, 73.4, 73.2, 72.8, 70.1, 68.2, 21.6. **HRMS (ESI)** calculated for C_34_H_35_O_6_S^+^ [M + H]^+^ 571.2154 found 571.2140.

#### 3.2.5. (3*S*,4*S*,9*R*,*E*)-9-(benzyloxy)-1-((*tert*-butyldimethylsilyl)oxy)-4-methyldec-5-en-3-ol (**5**)

To a solution of **14** (500 mg, 2.0 mmol, 1.0 equiv.) and alkene **13** (570 mg, 3.0 mmol, 1.5 equiv.) in DCM (40 mL, 0.05 M) at room temperature, the Grubbs II catalyst (340 mg, 0.4 mmol, 0.2 equiv.) was added. The reaction mixture was heated at 60 °C for 6 h before it was cooled back to room temperature and concentrated in vacuo. Purification of the crude product was performed via flash chromatography on silica (hexanes/EtOAc = 3/1) to afford **5** (658 mg, 81%) as a colorless oil. **TLC**: Rf = 0.3 (hexanes/EtOAc = 3/1), UV & PMA stain. $\alpha_{D}^{20}$ = −17.7 (c 1.2, CHCl_3_). **^1^H NMR** (500 MHz, CDCl_3_) δ 7.37–7.29 (m, 4H), 7.30–7.23 (m, 1H), 5.50–5.40 (m, 1H), 5.38–5.29 (m, 1H), 4.57 (d, *J* = 11.8 Hz, 1H), 4.44 (d, *J* = 11.7 Hz, 1H), 3.88 (dt, *J* = 9.9, 4.9 Hz, 1H), 3.78 (ddd, *J* = 10.0, 8.7, 3.9 Hz, 1H), 3.59 (ddd, *J* = 8.9, 6.4, 2.1 Hz, 1H), 3.51 (dt, *J* = 12.2, 6.2 Hz, 1H), 3.33–3.21 (m, 1H), 2.24–2.14 (m, 1H), 2.17–2.03 (m, 2H), 1.75–1.62 (m, 2H), 1.65–1.52 (m, 1H), 1.55–1.44 (m, 1H), 1.19 (d, *J* = 6.1 Hz, 3H), 1.02 (d, *J* = 6.8 Hz, 3H), 0.90 (s, 9H), 0.08 (s, 6H). **^13^C NMR** (125 MHz, CDCl_3_) δ 139.3, 133.0, 130.7, 128.4, 127.8, 127.5, 75.7, 74.5, 70.5, 63.0, 43.2, 36.7, 35.9, 28.9, 26.0, 19.7, 18.3, 16.2, −5.4, −5.4. **HRMS (ESI)** calculated for C_24_H_43_O_3_Si^+^ [M + H]^+^ 407.2981 found 407.2976.

#### 3.2.6. (3*S*,4*S*,9*R*,*E*)-9-(benzyloxy)-1-((*tert*-butyldimethylsilyl)oxy)-4-methyldec-5-en-3-yl N-(*tert*-butoxycarbonyl)-N-methyl-l-isoleucinate (**16**)

To a solution of alcohol **5** (300 mg, 0.74 mmol, 1.0 equiv.), acid **7** (272 mg, 1.11 mmol, 1.5 equiv.) and Et_3_N (0.41 mL, 2.96 mmol, 4.0 equiv.) in anhydrous PhMe (15 mL, 0.05 M) at 0 °C under an argon atmosphere was added to a solution of TCBC (0.35 mL, 2.22 mmol, 3.0 equiv.) in anhydrous PhMe (5 mL). The reaction mixture was stirred at room temperature for 30 min, and then a solution of DMAP (542 mg, 4.44 mmol, 6.0 equiv.) in PhMe (5 mL) was added. After being stirred at room temperature for 9 h, the reaction mixture was quenched with a saturated aqueous solution of NH_4_Cl (10 mL) and extracted with EtOAc (3 × 20 mL). The combined organic layers were washed with a saturated aqueous solution of brine (20 mL), dried over anhydrous Na_2_SO_4_, and then filtered and concentrated in vacuo. Purification of the crude product was performed via flash chromatography on silica gel (hexanes/EtOAc = 6/1) to afford **16** (356 mg, 76%) as a colorless oil. **TLC**: Rf = 0.4 (hexanes/EtOAc = 6/1), UV & PMA stain. ${\left[\alpha \right]}_{D}^{20}$= −51.8 (c 0.75, CHCl_3_). **^1^H NMR** (500 MHz, CDCl_3_) (mixture of conformers) δ 7.45–7.29 (m, 4H), 7.26 (dt, *J* = 7.5, 2.8 Hz, 1H), 5.55–5.37 (m, 1H), 5.32 (dd, *J* = 15.4, 7.5 Hz, 1H), 4.96–4.83 (m, 1H), 4.56 (d, *J* = 11.8 Hz, 1H), 4.51 (d, *J* = 8.0 Hz, 0.5H), 4.43 (d, *J* = 11.8 Hz, 1H), 4.27 (d, *J* = 10.7 Hz, 0.5H), 3.68–3.54 (m, 1H), 3.57–3.45 (m, 2H), 2.80 (d, *J* = 16.3 Hz, 3H), 2.38 (ddp, *J* = 22.6, 14.1, 6.9 Hz, 1H), 2.23–2.03 (m, 2H), 2.01–1.92 (m, 1H), 1.82–1.73 (m, 1H), 1.67 (dddd, *J* = 16.5, 9.3, 4.4, 1.7 Hz, 2H), 1.45 (s, 11H), 1.19 (dd, *J* = 6.4, 2.2 Hz, 3H), 1.07 (ddd, *J* = 13.8, 8.9, 7.1 Hz, 1H), 0.99–0.94 (m, 3H), 0.93–0.90 (m, 3H), 0.89–0.85 (m, 12H), 0.02 (s, 6H). **^13^C- NMR** (125 MHz, CDCl_3_) (mixture of conformers) δ 171.1, 170.6, 156.1, 155.6, 139.2, 133.7, 131.5, 131.2, 128.4, 128.0, 127.6, 127. 5, 80.1, 79.9, 75.1, 74.4, 70.4, 63.5, 62.3, 59.8, 59.5, 59.5, 40.5, 40.3, 39.7, 36.6, 34.7, 34.6, 33.3, 33.2, 30.2, 29.9, 28.8, 28.5, 26.0, 26.0, 24.9, 19.7, 19.5, 18.3, 18.3, 16.2, 16.0, 10.8, 10.3, −5.2, −5.3. **HRMS (ESI)** calculated for C_36_H_64_NO_6_Si^+^ [M + H]^+^ 634.4503 found 634.4531.

#### 3.2.7. (3*S*,4*S*,9*R*,*E*)-9-(benzyloxy)-1-((*tert*-butyldimethylsilyl)oxy)-4-methyldec-5-en-3-yl N-((*tert*-butoxycarbonyl)-d-phenylalanyl)-N-methyl-l-isoleucinate (**18**)

To a solution of **16** (300 mg, 0.47 mmol, 1.0 equiv.) in DCM (10 mL, 0.047 M), Et_3_N (0.46 mL, 3.29 mmol, 7.0 equiv.) at 0 °C was added, followed by the dropwise addition of trimethylsilyl trifluoromethanesulfonate (0.43 mL, 2.35 mmol, 5.0 equiv.). The reaction mixture was stirred for 3 h at room temperature and then quenched with a saturated aqueous solution of NaHCO_3_ (10 mL) and extracted with EtOAc (3 × 20 mL). The combined organic layers were washed with a saturated aqueous solution of NH_4_Cl (10 mL) and brine (10 mL), dried over anhydrous Na_2_SO_4_, and then filtered and concentrated in vacuo. The residue was used directly in the next step without further purification.

To a solution of crude amine (300 mg, 0.47 mmol, 1.0 equiv.) in DCM (10 mL, 0.047 M) at 0 °C, Boc-d-Phe-OH **6** was added (188 mg, 0.71 mmol, 1.5 equiv.), followed by an addition of DIPEA (0.25 mL, 1.41 mmol, 3.0 equiv.), HATU (357 mg, 0.94 mmol, 2.0 equiv.), and HOAt (97 mg, 0.71 mmol, 1.5 equiv.). The reaction mixture was stirred for 9 h at room temperature and then concentrated in vacuo, where the residue was redissolved in EtOAc (30 mL) and quenched with a 4% aqueous citric acid solution. The aqueous layer was extracted with EtOAc (3 × 20 mL) and the combined organic layers were washed with a saturated aqueous solution of NaHCO_3_ (15 mL) and brine (15 mL), dried over anhydrous Na_2_SO_4_, and then filtered and concentrated in vacuo. Purification of the crude product was performed via flash chromatography on silica gel (hexanes/EtOAc = 6/1) to afford **18** (224 mg, 61%) as a colorless oil. **TLC**: Rf = 0.4 (hexanes/EtOAc = 6/1), UV & PMA stain. ${\left[\alpha \right]}_{D}^{20}$= −46.5 (*c* 3.0, CHCl_3_). **^1^H NMR** (400 MHz, CDCl_3_) (mixture of conformers) δ 7.30 (dd, *J* = 5.6, 2.6 Hz, 4H), 7.26–7.12 (m, 6H), 5.38 (dt, *J* = 16.3, 6.6 Hz, 1H), 5.28 (dd, *J* = 13.7, 8.1 Hz, 1H), 5.17 (dd, *J* = 9.3, 3.4 Hz, 0.5H), 5.01 (dd, *J* = 11.6, 3.5 Hz, 0.5H), 4.89 (qd, *J* = 8.8, 7.4, 4.6 Hz, 1H), 4.77 (d, *J* = 10.7 Hz, 1H), 4.53 (d, *J* = 11.7 Hz, 1H), 4.41 (d, *J* = 11.8 Hz, 1H), 3.55 (ddd, *J* = 10.9, 6.9, 4.4 Hz, 1H), 3.51–3.40 (m, 2H), 3.03–2.89 (m, 2H), 2.85 (d, *J* = 11.9 Hz, 3H), 2.34 (q, *J* = 6.6 Hz, 1H), 2.05 (ddt, *J* = 16.5, 13.4, 5.7 Hz, 2H), 1.81–1.74 (m, 1H), 1.63 (dddd, *J* = 12.6, 9.3, 6.2, 1.4 Hz, 2H), 1.50–1.41 (m, 1H), 1.39–1.26 (m, 10H), 1.16 (dd, *J* = 6.2, 3.2 Hz, 3H), 0.98–0.80 (m, 17H), 0.73 (d, *J* = 3.6 Hz, 2H), 0.00–0.06 (m, 6H). **^13^C NMR** (100 MHz, CDCl_3_) (mixture of conformers) δ 172.9, 172.6, 170.5, 155.4, 154.9, 154.8, 139.2, 139.1, 136.7, 136.4, 131.6, 131.4, 131.3, 131.2, 131.1, 129.6, 129.5, 128.5, 128.4, 128.4, 127.7, 127.6, 127.6, 127.5, 126.9, 126.9, 126.7, 79.7, 76.1, 75.5, 74.4, 74.3, 65.4, 60.9, 60.5, 59.7, 59.4, 51.9, 51.8, 51.3, 40.2, 40.1, 39.2, 38.9, 36.5, 34.7, 34.4, 34.0, 33.8, 33.4, 31.3, 29.3, 28.8, 28.4, 28.3, 26.3, 26.0, 26.0, 25.1, 24.4, 19.7, 19.5, 18.3, 18.3, 16.0, 16.0, 14.8, 11.3, 10.8, −5.3, −5.3, −5.4. **HRMS (ESI)** calculated for C_45_H_73_N_2_O_7_Si^+^ [M + H]^+^ 781.5187 found 781.5182.

#### 3.2.8. (3*S*,4*S*,9*R*)-9-(benzyloxy)-1-((*tert*-butyldimethylsilyl)oxy)-4-methyldecan-3-yl N-((*tert*-butoxycarbonyl)-d-phenylalanyl)-N-methyl-l-isoleucinate (**19**)

To a solution of **18** (300 mg, 0.38 mmol, 1.0 equiv.) in EtOAc (5 mL, 0.076 M), palladium was added on charcoal (30 mg, 10% Pd, 0.013 mmol, 0.034 equiv.) under an argon atmosphere. The reaction flask was evacuated and purged with hydrogen three times. The reaction mixture was stirred under a hydrogen atmosphere at room temperature for 4 h, where the flask was then evacuated and purged with nitrogen three times and the catalyst was removed via filtration using a pad of celite. The filtrate was concentrated under reduced pressure to afford the crude product, which was purified via flash chromatography on silica gel (hexanes/EtOAc = 6/1) to afford **19** (268 mg, 90%) as a colorless oil. **TLC**: Rf = 0.4 (hexanes/EtOAc = 6/1), UV & PMA stain. ${\left[\alpha \right]}_{D}^{20}$= −43.2 (c 1.1, CDCl_3_). **^1^H NMR** (500 MHz, CDCl_3_) (mixture of conformers) δ 7.32 (dd, *J* = 7.3, 3.2 Hz, 4H), 7.29–7.11 (m, 6H), 5.29 (d, *J* = 9.0 Hz, 0.5H), 5.21 (d, *J* = 9.2 Hz, 0.5H), 5.01 (dq, *J* = 8.6, 5.1, 4.5 Hz, 1H), 4.91 (td, *J* = 8.5, 6.0 Hz, 0.5H), 4.87–4.75 (m, 0.5H), 4.55 (d, *J* = 11.8 Hz, 1H), 4.44 (d, *J* = 11.8 Hz, 1H), 3.59 (dt, *J* = 10.9, 5.6 Hz, 1H), 3.52–3.36 (m, 2H), 3.05–2.93 (m, 2H), 2.88 (d, *J* = 22.2 Hz, 3H), 1.78–1.62 (m, 3H), 1.57 (tt, *J* = 6.2, 3.4 Hz, 1H), 1.39 (s, 6H), 1.36–1.25 (m, 9H), 1.24–1.07 (m, 4H), 1.01–0.79 (m, 18H), 0.76 (d, J = 4.0 Hz, 2H), 0.14–0.30 (m, 6H). **^13^C NMR** (125 MHz, CDCl_3_) (mixture of conformers) δ 172.7, 170.6, 154.9, 139.3, 136.5, 129.6, 129.5, 128.5, 128.3, 127.6, 127.4, 127.4, 126.8, 126.7, 79.6, 75.9, 75.3, 74.9, 74.9, 70.4, 65.4, 60.9, 60.7, 59.8, 59.6, 53.5, 51.9, 51.2, 40.1, 36.7, 36.4, 34.7, 34.3, 34.1, 33.4, 33.1, 33.0, 32.8, 31.3, 29.3, 28.4, 28.3, 27.4, 26.0, 25.9, 25.8, 25.1, 19.7, 19.7, 18.2, 15.8, 15.8, 14.7, 14.6, 11.3, 10.8, −5.3, −5.4. **HRMS (ESI)** calculated for C_45_H_75_N_2_O_7_Si^+^ [M + H]^+^ 783.5344 found 783.5338.

#### 3.2.9. (3*S*,4*S*,9*R*)-9-(benzyloxy)-1-hydroxy-4-methyldecan-3-yl N-((*tert*-butoxycarbonyl)-d-phenylalanyl)-N-methyl-l-isoleucinate (**20**)

To a solution of **19** (250 mg, 0.32 mmol, 1.0 equiv.) in MeOH (5 mL, 0.064 M) at 0 °C, CSA (15 mg, 0.064 mmol, 0.2 equiv.) was added. The reaction mixture was stirred for 3 h at room temperature and then concentrated in vacuo, and the oily residue was redissolved in EtOAc (30 mL) and washed with a saturated aqueous solution of NaHCO_3_ (15 mL) and brine (15 mL), dried over anhydrous Na_2_SO_4_, and then filtered and concentrated in vacuo. Purification of the crude product was performed via flash chromatography on silica gel (hexanes/EtOAc = 3/1) to afford **20** (167 mg, 78%) as a colorless oil. **TLC**: Rf = 0.4 (hexanes/EtOAc = 3/1), UV & PMA stain. ${\left[\alpha \right]}_{D}^{20}$= −52.5 (c 2.9, CHCl_3_). **^1^H NMR** (500 MHz, CDCl_3_) (mixture of conformers) δ 7.37–7.28 (m, 4H), 7.28–7.12 (m, 6H), 5.36 (d, *J* = 9.0 Hz, 1H), 5.07–4.92 (m, 1H), 4.87 (q, *J* = 8.0 Hz, 1H), 4.82 (d, *J* = 10.9 Hz, 1H), 4.53 (d, *J* = 11.8 Hz, 1H), 4.42 (d, *J* = 11.8 Hz, 1H), 3.52 (dt, J = 10.9, 5.4 Hz, 1H), 3.46 (ddt, *J* = 12.2, 8.7, 4.0 Hz, 2H), 3.03–2.89 (m, 2H), 2.88 (d, *J* = 9.4 Hz, 3H), 1.84 (q, J = 8.4 Hz, 1H), 1.71 (h, *J* = 4.8, 4.0 Hz, 2H), 1.65–1.50 (m, 2H), 1.38 (s, 9H), 1.33 (d, *J* = 10.3 Hz, 6H), 1.22–1.06 (m, 4H), 0.95–0.78 (m, 9H), 0.77–0.70 (m, 2H).**^13^C NMR** (125 MHz, CDCl_3_) (mixture of conformers) δ 172.9, 171.1, 155.3, 139.1, 136.3, 129.5, 129.5, 129.4, 128.5, 128.4, 128.3, 127.6, 127.6, 127.5, 127.3, 126.8, 126.6, 79.9, 75.5, 74.8, 74.8, 70.3, 65.1, 60.8, 58.9, 58.6, 51.9, 51.2, 39.4, 39.0, 36.7, 36.6, 34.6, 34.2, 33.2, 32.8, 32.7, 30.9, 29.2, 28.3, 28.3, 27.3, 27.2, 26.3, 25.7, 24.7, 19.6, 15.7, 14.8, 14.6, 10.6. **HRMS (ESI)** calculated for C_39_H_61_N_2_O_7_^+^ [M + H]^+^ 669.4479 found 669.4483.

#### 3.2.10. (3*S*,4*R*,6*S*,7*S*,12*R*)-12-(benzyloxy)-4-hydroxy-3,7-dimethyltridec-1-en-6-yl N-((*tert*-butoxycarbonyl)-d-phenylalanyl)-N-methyl-l-isoleucinate (**21**)

To a solution of alcohol **20** (235 mg, 0.35 mmol, 1.0 equiv.) in dry DCM (10 mL, 0.035 M) at 0 °C under argon atmosphere, Dess–Martin periodinane was added (225 mg, 0.53 mmol, 1.5 equiv.). After being stirred at room temperature for 4 h, the reaction mixture was quenched with a saturated aqueous solution of Na_2_SO_3_ (10 mL) and extracted with EtOAc (3 × 20 mL). The combined organic extracts were washed with a saturated aqueous solution of copper sulfate (15 mL) and brine (15 mL), dried over anhydrous Na_2_SO_4_, and then filtered and concentrated in vacuo. The residue was used directly in the next step without further purification.

To a suspension of *t*BuOK (196 mg, 1.75 mmol, 5.0 equiv.) in anhydrous THF (10 mL, 0.035 M) at −78 °C, *trans*-2-butene was added (137 mg, 2.45 mmol, 7.0 equiv.), followed by the dropwise addition of *n*BuLi (1.1 mL, 1.75 mmol, 1.6 M in hexane, 5.0 equiv.). After the addition of *n*BuLi, the reaction mixture was warmed to −45 °C and stirred for 30 min. After being recooled to −78 °C, a solution of (-)-Ipc_2_BOMe (664 mg, 2.1 mmol, 6.0 equiv.) in anhydrous THF (5 mL) was added and stirred for 30 min at −78 °C. BF_3_·Et_2_O (0.35 mL, 2.8 mmol, 8.0 equiv.) was then added, followed by a solution of the resulting aldehyde (235 mg, 0.35 mmol, 1.0 equiv.) in THF (5 mL). After being stirred at −78 °C for 10 h, the reaction mixture was quenched with MeOH (10 mL) and warmed to room temperature. The solvent was removed in vacuo and the residue was redissolved in THF (20 mL) and H_2_O (6 mL), and NaBO_3_.4H_2_O (377 mg, 2.45 mmol, 7.0 equiv.) was then added. The mixture was stirred at room temperature for 12 h and poured into a mixture of EtOAc (30 mL) and H_2_O (3 mL). The layers were separated and the aqueous layer was further extracted with EtOAc (2 × 20 mL). The combined organic extracts were washed with brine (10 mL), dried over anhydrous Na_2_SO_4_, and then filtered and concentrated in vacuo. Purification of the crude product was performed via flash chromatography on silica (hexanes/EtOAc = 3/1) to afford two separable diastereoisomers (180 mg, 71% total yield, dr > 20:1) with **21** (colorless oil) as the major product. **TLC**: Rf = 0.3 (hexanes/EtOAc = 3/1), UV & PMA stain. ${\left[\alpha \right]}_{D}^{20}$= −30.81 (c 0.57, CHCl_3_). **^1^H NMR** (500 MHz, CDCl_3_) (mixture of conformers) δ 7.32 (dd, *J* = 6.4, 3.8 Hz, 4H), 7.29–7.14 (m, 6H), 5.90–5.69 (m, 1H), 5.44 (d, *J* = 8.8 Hz, 0.5H), 5.20 (dd, *J* = 9.2, 6.5 Hz, 0.5H), 5.15–5.04 (m, 2H), 5.02 (q, *J* = 1.5 Hz, 1H), 5.00–4.94 (m, 1H), 4.88–4.75 (m, 1H), 4.55 (d, *J* = 11.8 Hz, 1H), 4.44 (d, *J* = 11.8 Hz, 1H), 3.48 (h, *J* = 6.0 Hz, 1H), 3.45–3.38 (m, 1H), 3.08–2.92 (m, 2H), 2.89 (d, *J* = 22.8 Hz, 3H), 2.31–2.19 (m, 1H), 1.90–1.82 (m, 1H), 1.70–1.60 (m, 2H), 1.57 (ddd, *J* = 12.3, 6.5, 2.3 Hz, 2H), 1.40 (s, 9H), 1.38–1.24 (m, 6H), 1.16 (dd, *J* = 16.8, 6.1 Hz, 3H), 1.11–1.00 (m, 4H), 0.98–0.83 (m, 9H), 0.82–0.69 (m, 2H). **^13^C NMR** (125 MHz, CDCl_3_) (mixture of conformers) δ 172.9, 170.8, 155.4, 139.9, 139.7, 139.3, 129.7, 129.6, 129.5, 129.5, 128.5, 128.4, 126.8, 116.6, 115.9, 79.9, 77.3, 75.0, 74.9, 74.7, 73.1, 72.6, 70.4, 61.0, 60.9, 52.4, 44.1, 43.8, 39.5, 36.9, 36.8, 36.3, 33.9, 33.5, 32.8, 32.7, 31.6, 30.9, 28.5, 28.4, 27.5, 25.8, 24.8, 19.8, 19.7, 16.2, 16.1, 15.7, 14.7, 14.2, 10.6. **HRMS (ESI)** calculated for C_43_H_67_N_2_O_7_^+^ [M + H]^+^ 723.4948 found 723.4943.

#### 3.2.11. (3*S*,4*R*,6*S*,7*S*,12*R*)-12-(benzyloxy)-4-((tert-butyldimethylsilyl)oxy)-3,7-dimethyltridec-1-en-6-yl N-((*tert*-butoxycarbonyl)-d-phenylalanyl)-N-methyl-l-isoleucinate (**22**)

To a solution of alcohol **21** (230 mg, 0.32 mmol, 1.0 equiv.) in dry DCM (5 mL, 0.064 M) at 0 °C under an argon atmosphere, Et_3_N was added (89 μL, 0.64 mmol, 2.0 equiv.), followed by TBSOTf (110 μL, 0.48 mmol, 1.5 equiv.). The reaction mixture was warmed up to an ambient temperature, stirred for 2 h, and then quenched with a saturated aqueous solution of NaHCO_3_ (5 mL). The aqueous layer was extracted with EtOAc (2 × 20 mL). The combined organic layers were washed with H_2_O (10 mL) and brine (10 mL), dried over anhydrous Na_2_SO_4_, and then filtered and concentrated in vacuo. Purification of the crude product was performed via flash chromatography on silica (hexanes/EtOAc = 9/1) to afford silyl ether **22** (217 mg, 81%) as a colorless oil. **TLC**: Rf = 0.5 (hexanes/EtOAc = 6/1), UV & PMA stain. ${\left[\alpha \right]}_{D}^{20}$= −19.33 (c 0.73, CHCl_3_). **^1^H NMR** (500 MHz, CDCl_3_) (mixture of conformers) δ 7.37–7.29 (m, 4H), 7.29–7.13 (m, 6H), 5.85–5.61 (m, 1H), 5.33–5.25 (m, 0.5H), 5.19 (t, *J* = 9.5 Hz, 0.5H), 5.01 (ddt, *J* = 13.7, 8.4, 2.5 Hz, 2H), 4.98–4.89 (m, 2H), 4.83 (dd, *J* = 12.9, 6.7 Hz, 1H), 4.60–4.52 (m, 1H), 4.48–4.40 (m, 1H), 3.54 (dtd, *J* = 13.9, 7.3, 6.6, 3.0 Hz, 1H), 3.52–3.39 (m, 1H), 3.07–2.91 (m, 2H), 2.88 (s, 3H), 2.42–2.24 (m, 1H), 1.70–1.60 (m, 2H), 1.57 (ddt, *J* = 8.3, 6.5, 4.2 Hz, 2H), 1.38 (d, *J* = 1.3 Hz, 9H), 1.36–1.24 (m, 6H), 1.18 (dd, *J* = 6.1, 2.5 Hz, 3H), 0.96 (d, *J* = 6.9 Hz, 4H), 0.93–0.85 (m, 14H), 0.86–0.79 (m, 4H), 0.78 (td, *J* = 6.3, 2.5 Hz, 2H), 0.13–0.01 (m, 6H). **^13^C NMR** (125 MHz, CDCl_3_) (mixture of conformers) δ 173.0, 172.7, 170.8, 170.6, 155.5, 154.9, 140.1, 140.0, 136.6, 129.7, 129.5, 128.6, 128.5, 128.4, 127.7, 127.7, 127.5, 126.9, 126.9, 126.8, 115.3, 79.7, 76.2, 75.3, 75.3, 75.0, 75.0, 74.8, 72.7, 72.5, 70.4, 61.0, 60.8, 52.0, 51.9, 42.3, 42.2, 40.4, 39.0, 37.0, 36.8, 36.8, 36.7, 36.3, 33.8, 33.7, 33.1, 32.8, 31.3, 31.3, 28.5, 28.4, 27.6, 26.5, 26.1, 26.0, 26.0, 26.0, 25.9, 25.3, 24.5, 23.3, 19.8, 19.7, 18.2, 18.2, 17.4, 17.3, 15.9, 14.7, 14.5, 14.4, 11.4, 10.9, −4.1, −4.2, −4.3, −4.5, −4.5. **HRMS (ESI)** calculated for C_49_H_81_N_2_O_7_Si^+^ [M + H]^+^ 837.5813 found 837.5814.

#### 3.2.12. (6*R*,9*S*,12*S*,14*R*,15*R*)-6-benzyl-12-((2*S*,7*R*)-7-(benzyloxy)octan-2-yl)-9-((*S*)-*sec*-butyl)-14-((*tert*-butyldimethylsilyl)oxy)-2,2,8,15-tetramethyl-4,7,10-trioxo-3,11-dioxa-5,8-diazahexadecan-16-oic acid (**23**)

To a solution of alkene **22** (215 mg, 0.26 mmol, 1.0 equiv.) in DCM at –78 °C, ozone was bubbled through the solution until the solution became slightly blue. PPh_3_ (341 mg, 1.3 mmol, 5.0 equiv.) was added and the resultant solution was stirred at room temperature for 5 h. After concentration, the residue was used directly in the next step without further purification.

To a solution of the above crude aldehyde and PhI(OAc)_2_ (251 mg, 0.78 mmol, 3.0 equiv.) in DCM (5 mL, 0.052 M) at 0 °C, TEMPO (41 mg, 0.26 mmol, 1.0 equiv.) and H_2_O (1 mL) were added. The reaction mixture was warmed up to an ambient temperature, stirred for 12 h, and then quenched with a saturated aqueous solution of Na_2_SO_3_ (5 mL). The aqueous layer was extracted with DCM (2 × 20 mL) and the combined organic layers were washed with H_2_O (10 mL) and brine (10 mL), dried over anhydrous Na_2_SO_4_, and then filtered and concentrated in vacuo. Purification of the crude product was performed via flash chromatography on silica (hexanes/EtOAc = 1/1) to afford acid **23** (149 mg, 68%) as a colorless oil. **TLC**: Rf = 0.4 (hexanes/EtOAc = 3/1), UV & PMA stain. ${\left[\alpha \right]}_{D}^{20}$= −32.07 (c 0.92, CHCl_3_). **^1^H NMR** (500 MHz, CDCl_3_) (mixture of conformers) δ 8.03 (dd, *J* = 8.0, 6.4 Hz, 1H), 7.37–7.29 (m, 4H), 7.21 (d, *J* = 5.9 Hz, 6H), 5.35 (d, *J* = 9.6 Hz, 1H), 5.13 (dq, J = 12.5, 6.3 Hz, 1H), 4.93 (td, *J* = 8.5, 6.4 Hz, 1H), 4.74 (t, *J* = 9.9 Hz, 1H), 4.56 (d, *J* = 11.8 Hz, 1H), 4.45 (d, *J* = 11.8 Hz, 1H), 3.97–3.82 (m, 1H), 3.49 (q, *J* = 6.0 Hz, 1H), 3.10–2.94 (m, 2H), 2.93 (s, 3H), 2.75 (t, *J* = 5.8 Hz, 1H), 1.78–1.65 (m, 2H), 1.59 (ddd, *J* = 15.0, 9.7, 5.8 Hz,2H), 1.38 (d, J = 2.2 Hz, 9H), 1.37–1.26 (m, 6H), 1.24–1.15 (m, 2H), 1.15 (dq, *J* = 8.9, 5.6, 4.2 Hz, 3H), 1.08 (dd, *J* = 9.1, 6.1 Hz, 2H), 0.95–0.84 (m, 18H), 0.78 (ddd, *J* = 11.8, 7.4, 5.1 Hz, 2H), 0.10 (d, *J* = 4.3 Hz, 6H). **^13^C NMR** (125 MHz, CDCl_3_) (mixture of conformers) δ 176.5, 173.0, 170.9, 170.4, 166.2, 154.9, 139.2, 137.6, 136.4, 132.7, 131.0, 130.3, 129.6, 129.4, 128.6, 128.5, 128.5, 128.3, 127.7, 127.6, 127.4, 126.9, 126.8, 79.8, 75.2, 75.0, 71.6, 71.5, 70.4, 61.0, 52.0, 44.2, 44.0, 40.2, 36.7, 36.6, 36.1, 35.4, 33.6, 32.5, 31.3, 28.4, 28.3, 27.5, 27.3, 26.6, 25.8, 25.8, 25.4, 20.1, 20.1, 19.7, 19.7, 17.9, 15.8, 14.7, 14.1, 14.0, 11.3, 10.7, 10.7, −4.3, −4.8, −4.9. **HRMS (ESI)** calculated for C_48_H_79_N_2_O_9_Si^+^ [M + H]^+^ 855.5555 found 855.5557.

#### 3.2.13. (3*S*,6*R*,9*R*,10*R*,12*S*)-6-benzyl-12-((2*S*,7*R*)-7-(benzyloxy)octan-2-yl)-3-((*S*)-*sec*-butyl)-10-((*tert*-butyldimethylsilyl)oxy)-4,9-dimethyl-1-oxa-4,7-diazacyclododecane-2,5,8-trione (**24**)

To a solution of **23** (150 mg, 0.18 mmol, 1.0 equiv.) in DCM (5 mL, 0.036 M) at 0 °C, Et_3_N (0.18 mL, 1.26 mmol, 7.0 equiv.) was added, followed by the dropwise addition of trimethylsilyl trifluoromethanesulfonate (0.16 mL, 0.90 mmol, 5.0 equiv.). The reaction mixture was stirred for 6 h at room temperature and then quenched with a saturated aqueous solution of NaHCO_3_ (5 mL) and extracted with EtOAc (2 × 20 mL). The combined organic layers were washed with a saturated aqueous solution of NH_4_Cl (10 mL) and brine (10 mL), dried over anhydrous Na_2_SO_4_, and then filtered and concentrated in vacuo. The residue was used directly in the next step without further purification.

To a solution of above crude amine in DCM (120 mL, 0.0015 M) at 0 °C, DIPEA (0.31 mL, 1.8 mmol, 10.0 equiv.), HATU (342 mg, 0.90 mmol, 5.0 equiv.), and HOAt was added (73 mg, 0.54 mmol, 3.0 equiv.). The reaction mixture was stirred for 9 h at room temperature and then concentrated in vacuo, where the residue was redissolved in EtOAc (30 mL) and quenched with a 4% aqueous citric acid solution. The aqueous layer was extracted with EtOAc (3 × 20 mL) and the combined organic layers were washed with a saturated aqueous solution of NaHCO_3_ (15 mL) and brine (15 mL), dried over anhydrous Na_2_SO_4_, and then filtered and concentrated in vacuo. Purification of the crude product was performed via flash chromatography on silica gel (hexanes/EtOAc = 2/1) to afford **24** (68 mg, 51%) as a white solid. **TLC**: Rf = 0.3 (hexanes/EtOAc = 1/1), UV & PMA stain. ${\left[\alpha \right]}_{D}^{20}$= +7.71 (c 0.64, CHCl_3_). **^1^H NMR** (500 MHz, CDCl_3_) δ 7.42–7.29 (m, 4H), 7.29–7.16 (m, 6H), 5.76 (d, *J* = 10.3 Hz, 1H), 5.23 (td, *J* = 10.9, 4.5 Hz, 1H), 4.93 (d, *J* = 11.7 Hz, 1H), 4.76 (ddd, *J* = 11.0, 5.2, 1.7 Hz, 1H), 4.55 (d, *J* = 11.7 Hz, 1H), 4.45 (d, *J* = 11.8 Hz, 1H), 4.17 (dd, *J* = 8.0, 3.2 Hz, 1H), 3.48 (q, *J* = 5.9 Hz, 1H), 3.22 (t, *J* = 12.0 Hz, 1H), 2.91 (dd, *J* = 12.5, 4.5 Hz, 1H), 2.56 (s, 3H), 2.52 (dd, *J* = 6.7, 3.2 Hz, 1H), 1.92 (dt, *J* = 7.6, 4.0 Hz, 1H), 1.86 (ddd, *J* = 11.3, 6.6, 3.8 Hz, 1H), 1.79–1.68 (m, 1H), 1.60 (dt, *J* = 15.5, 5.0 Hz, 2H), 1.46–1.35 (m, 3H), 1.32–1.22 (m, 2H), 1.18 (d, *J* = 6.1 Hz, 5H), 1.07 (d, *J* = 6.6 Hz, 3H), 0.92 (d, *J* = 3.2 Hz, 9H), 0.87 (d, *J* = 6.4 Hz, 5H), 0.77–0.66 (m, 6H), 0.22 (s, 3H), 0.15 (s, 3H). **^13^C NMR** (125 MHz, CDCl_3_) δ 174.5, 174.0, 168.9, 139.2, 136.6, 129.3, 128.7, 128.4, 127.7, 127.4, 127.0, 76.1, 75.0, 70.4, 68.9, 60.6, 50.9, 48.3, 37.3, 36.8, 33.8, 32.7, 32.2, 30.5, 30.1, 26.5, 26.0, 25.7, 23.7, 19.7, 18.2, 15.6, 15.4, 10.0, 7.6, −3.7, −3.8. **HRMS (ESI)** calculated for C_43_H_69_N_2_O_6_Si^+^ [M + H]^+^ 737.4925 found 737.4924.

#### 3.2.14. (3*S*,6*R*,9*R*,10*R*,12*S*)-6-benzyl-3-((*S*)-*sec*-butyl)-10-((*tert*-butyldimethylsilyl)oxy)-12-((2*S*,7*R*)-7-hydroxyoctan-2-yl)-4,9-dimethyl-1-oxa-4,7-diazacyclododecane-2,5,8-trione (**4**)

To a solution of **24** (65 mg, 0.088 mmol, 1.0 equiv.) in EtOAc (3 mL, 0.029 M) under an argon atmosphere, palladium was added on charcoal (20 mg, 10% Pd, 0.0085 mmol, 0.1 equiv.). The reaction flask was evacuated and purged with hydrogen three times. The reaction mixture was stirred under a hydrogen atmosphere at room temperature for 10 h; the flask was then evacuated and purged with nitrogen three times and the catalyst was removed via filtration using a pad of celite. The filtrate was concentrated under reduced pressure to afford the crude product, which was purified via flash chromatography on silica gel (hexanes/EtOAc = 1/1) to afford **4** (52 mg, 91%) as a white solid. **TLC**: Rf = 0.3 (hexanes/EtOAc = 2/1), UV & PMA stain.${\left[\alpha \right]}_{D}^{20}$= −62.2 (c 0.52, CDCl_3_). **^1^H NMR** (400 MHz, CDCl_3_) δ 7.57–6.93 (m, 5H), 5.82 (d, *J* = 10.3 Hz, 1H), 5.24 (td, *J* = 11.0, 4.6 Hz, 1H), 4.93 (d, *J* = 11.7 Hz, 1H), 4.91–4.69 (m, 1H), 4.17 (dd, *J* = 7.8, 3.1 Hz, 1H), 3.88–3.68 (m, 1H), 3.23 (t, *J* = 12.0 Hz, 1H), 2.92 (dd, *J* = 12.5, 4.6 Hz, 1H), 2.58 (s, 3H), 2.53 (dd, *J* = 6.7, 3.2 Hz, 1H), 1.94 (td, *J* = 7.2, 3.5 Hz, 1H), 1.91–1.82 (m, 1H), 1.75 (ddd, *J* = 15.8, 7.9, 1.8 Hz, 1H), 1.62 (dd, *J* = 15.7, 5.1 Hz, 2H), 1.43 (ddd, *J* = 17.7, 10.7, 4.2 Hz, 3H), 1.36–1.24 (m, 3H), 1.19 (d, *J* = 6.1 Hz, 5H), 1.08 (d, *J* = 6.7 Hz, 3H), 0.93 (s, 9H), 0.88 (d, *J* = 6.4 Hz, 3H), 0.86–0.68 (m, 7H), 0.23 (s, 3H), 0.16 (s, 3H). **^13^C NMR** (100 MHz, CDCl_3_) δ 174.6, 174.1, 169.0, 136.6, 129.3, 128.7, 127.1, 76.1, 69.0, 68.2, 60.7, 51.0, 48.3, 39.5, 37.3, 33.8, 32.7, 32.3, 30.6, 30.2, 26.8, 26.1, 25.8, 23.8, 23.7, 18.2, 15.7, 15.5, 10.1, 7.6, −3.6, −3.8. **HRMS (ESI)** calculated for C_36_H_63_N_2_O_6_Si^+^ [M + H]^+^ 647.4455 found 647.4448.

#### 3.2.15. (3*S*,6*R*,9*R*,10*R*,12*S*)-6-benzyl-12-((2*S*,7*R*)-7-(((2*R*,4a*R*,6*R*,7*S*,8*S*,8a*R*)-7,8-bis(benzyloxy)-2-phenylhexahydropyrano [3,2-d][1,3]dioxin-6-yl)oxy)octan-2-yl)-3-((*S*)-*sec*-butyl)-10-((*tert*-butyldimethylsilyl)oxy)-4,9-dimethyl-1-oxa-4,7-diazacyclododecane-2,5,8-trione (**25**)

Glycosyl donor **3** (55 mg, 0.096 mmol, 1.2 equiv.) and 2,6-*tert*-butyl-4-methyl pyridine (43 mg, 0.21 mmol, 2.6 equiv.) were dried under a high vacuum for 1 h prior to being dissolved in DCM (5 mL, 0.016M). After a 4Å Molecular Sieve (200 mg, powder) was added, the suspension was stirred at room temperature for 1 h and then cooled to –78 °C. Tf_2_O (17 μL, 0.10 mmol, 1.3 equiv.) was added at –78 °C, and, 10 min later, a solution of glycosyl acceptor **4** (52 mg, 0.080 mmol, 1.0 equiv.) in DCM (2 mL) was dropwise added at –78 °C. The reaction mixture was stirred at –78 °C for 4 h and then quenched with water (3 mL). The reaction mixture was filtered, and the filtrate was diluted with EtOAc (30 mL), washed with H_2_O (10 mL) and brine (10 mL), dried over anhydrous Na_2_SO_4_, and then filtered and concentrated in vacuo. Purification of the crude product was performed via flash chromatography on silica (hexanes/EtOAc = 6/1) to afford **25** (53 mg, 61%) as a white solid. **TLC**: Rf = 0.4 (hexanes/EtOAc = 6/1), UV & PMA stain.${\left[\alpha \right]}_{D}^{20}$= −57.67 (c 0.57, CHCl_3_). **^1^H NMR** (500 MHz, CDCl_3_) δ 7.63–7.45 (m, 4H), 7.43–7.06 (m, 16H), 5.82 (d, *J* = 10.4 Hz, 1H), 5.62 (s, 1H), 5.24 (td, *J* = 10.9, 4.5 Hz, 1H), 4.99 (d, *J* = 12.4 Hz, 1H), 4.93 (d, *J* = 11.7 Hz, 1H), 4.90–4.83 (m, 1H), 4.83–4.75 (m, 1H), 4.72–4.65 (m, 1H), 4.63–4.57 (m, 1H), 4.54 (s, 1H), 4.29 (dd, *J* = 10.5, 4.8 Hz, 1H), 4.25–4.19 (m, 1H), 4.18 (dd, *J* = 8.0, 3.1 Hz, 1H), 3.95 (t, *J* = 10.3 Hz, 1H), 3.90–3.78 (m, 2H), 3.60 (dd, *J* = 9.9, 3.0 Hz, 1H), 3.32 (td, *J* = 9.7, 4.9 Hz, 1H), 3.23 (t, *J* = 12.0 Hz, 1H), 2.92 (dd, *J* = 12.6, 4.6 Hz, 1H), 2.58 (s, 3H), 2.53 (dd, *J* = 6.7, 3.1 Hz, 1H), 2.00–1.91 (m, 1H), 1.87 (dt, *J* = 11.8, 6.7 Hz, 1H), 1.75 (dd, *J* = 15.8, 7.8 Hz, 1H), 1.63 (dd, *J* = 15.5, 5.3 Hz, 2H), 1.45 (dt, *J* = 16.8, 8.1 Hz, 2H), 1.41–1.28 (m, 3H), 1.25–1.16 (m, 3H), 1.12 (d, *J* = 6.1 Hz, 2H), 1.08 (dd, *J* = 6.7, 3.4 Hz, 4H), 0.93 (s, 9H), 0.88 (d, *J* = 6.4 Hz, 3H), 0.81–0.65 (m, 7H), 0.23 (s, 3H), 0.16 (s, 3H). **^13^C NMR** (125 MHz, CDCl_3_) δ 174.7, 174.1, 173.5, 169.0, 138.9, 138.7, 137.9, 136.7, 129.4, 129.0, 128.7, 128.7, 128.4, 128.3, 128.2, 127.7, 127.6, 127.5, 127.0, 126.4, 126.3, 101.6, 99.7, 78.9, 78.4, 76.9, 76.1, 74.9, 74.2, 72.5, 69.0, 68.9, 67.9, 60.8, 51.1, 48.4, 37.4, 33.9, 32.7, 32.3, 30.6, 30.1, 29.9, 26.3, 26.1, 25.6, 23.8, 19.2, 18.2, 15.8, 15.6, 10.0, 7.6, −3.7, −3.8. **HRMS (ESI)** calculated for C_63_H_89_N_2_O_11_Si^+^ [M + H]^+^ 1077.6236 found 1077.6238.

#### 3.2.16. (3*S*,6*R*,9*R*,10*R*,12*S*)-6-benzyl-12-((2*S*,7*R*)-7-(((2*R*,4a*R*,6*R*,7*S*,8*S*,8a*R*)-7,8-bis(benzyloxy)-2-phenylhexahydropyrano [3,2-d][1,3]dioxin-6-yl)oxy)octan-2-yl)-3-((*S*)-*sec*-butyl)-10-hydroxy-4,9-dimethyl-1-oxa-4,7-diazacyclododecane-2,5,8-trione (**26**)

To a solution of **25** (52 mg, 0.048 mmol, 1.0 equiv.) in THF (3 mL, 0.016 M), TBAF was added (0.58 mL, 0.58 mmol, 12.0 equiv., 1.0 M in THF) at 0 °C. The reaction mixture was warmed up to an ambient temperature, stirred for 6 h, and then quenched with a saturated aqueous solution of NH_4_Cl (3 mL). The aqueous layer was extracted with EtOAc (2 × 20 mL). The combined organic layers were washed with H_2_O (10 mL) and brine (10 mL), dried over anhydrous Na_2_SO_4_, and then filtered and concentrated in vacuo. Purification of the crude product was performed via flash chromatography on silica (hexanes/EtOAc = 3/1) to afford alcohol **26** (29 mg, 63%) as a colorless oil. **TLC**: Rf = 0.3 (hexanes/EtOAc = 3/1), UV & PMA stain. ${\left[\alpha \right]}_{D}^{20}$= −32.07 (c 0.92, CHCl_3_). **^1^H NMR** (400 MHz, CDCl_3_) δ 7.66–7.44 (m, 4H), 7.44–7.11 (m, 16H), 6.52 (d, *J* = 10.2 Hz, 1H), 5.74 (s, 1H), 5.29 (td, *J* = 10.8, 4.7 Hz, 1H), 5.00 (d, *J* = 12.3 Hz, 1H), 4.91 (t, *J* = 12.4 Hz, 2H), 4.82 (ddd, *J* = 10.7, 4.4, 2.0 Hz, 1H), 4.72 (d, *J* = 12.5 Hz, 1H), 4.63 (d, *J* = 12.6 Hz, 1H), 4.56 (s, 1H), 4.31 (dd, *J* = 10.4, 4.8 Hz, 1H), 4.25 (t, *J* = 9.6 Hz, 1H), 4.20–4.10 (m, 1H), 4.00 (t, *J* = 10.3 Hz, 1H), 3.92–3.79 (m, 2H), 3.63 (dd, *J* = 9.9, 3.2 Hz, 1H), 3.34 (td, *J* = 9.8, 4.9 Hz, 1H), 3.23 (t, *J* = 11.9 Hz, 1H), 2.93 (dd, *J* = 12.6, 4.7 Hz, 1H), 2.67 (s, 3H), 2.53 (dd, *J* = 6.8, 3.3 Hz, 1H), 2.00 (qd, *J* = 9.7, 7.9, 2.9 Hz, 1H), 1.90 (dt, *J* = 11.5, 7.1 Hz, 1H), 1.82–1.56 (m, 3H), 1.54–1.37 (m, 3H), 1.39–1.28 (m, 2H), 1.28 (s, 3H), 1.11 (dd, *J* = 23.9, 6.4 Hz, 6H), 0.89 (d, *J* = 6.3 Hz, 4H), 0.85–0.70 (m, 6H). **^13^C NMR** (100 MHz, CDCl_3_) δ 174.5, 174.0, 168.8, 138.6, 138.5, 137.8, 136.7, 129.3, 128.9, 128.7, 128.7, 128.7, 128.4, 128.3, 128.3, 127.7, 127.6, 127.0, 126.2, 101.5, 99.8, 78.8, 78.2, 76.4, 75.0, 74.6, 72.4, 68.8, 68.2, 67.8, 60.8, 50.7, 48.3, 37.2, 34.1, 32.0, 31.9, 30.5, 30.3, 29.8, 26.2, 25.3, 23.8, 19.5, 15.9, 15.6, 10.1, 7.7. **HRMS (ESI)** calculated for C_57_H_75_N_2_O_11_^+^ [M + H]^+^ 963.5371 found 963.5371.

#### 3.2.17. Colletotrichamide A

To a solution of **26** (25 mg, 0.026 mmol, 1.0 equiv.) in MeOH (3 mL, 0.009 M), palladium was added on charcoal (25 mg, 10% Pd, 0.011 mmol, 0.423 equiv.) under an argon atmosphere. The reaction flask was evacuated and purged with hydrogen three times. The reaction mixture was stirred under a hydrogen atmosphere at room temperature for 12 h, where the flask was then evacuated and purged with nitrogen three times and the catalyst was removed via filtration using a pad of celite. The filtrate was concentrated under reduced pressure to afford the crude product, which was performed via flash chromatography on silica gel (MeOH/DCM = 1/10) to afford colletotrichamide A (15 mg, 83%) as a colorless amorphous solid. **TLC**: Rf = 0.3 (MeOH/DCM = 1/10), UV & PMA stain.${\left[\alpha \right]}_{D}^{25}$= −78.10 (c 1.0, MeOH). **^1^H NMR** (400 MHz, DMSO) δ 8.58 (d, *J* = 9.5 Hz, 1H), 7.26 (d, *J* = 7.1 Hz, 2H), 7.23 (d, *J* = 4.0 Hz, 2H), 7.23–7.14 (m, 1H), 5.06 (dt, *J* = 9.4, 6.2 Hz, 1H), 4.77 (d, *J* = 11.6 Hz, 1H), 4.71 (s, 1H), 4.64 (d, *J* = 10.0 Hz, 1H), 4.53 (s, 1H), 4.50 (d, *J* = 5.1 Hz, 1H), 4.43 (s, 1H), 4.35 (t, *J* = 5.5 Hz, 1H), 4.17 (d, *J* = 4.3 Hz, 1H), 3.88 (d, *J* = 8.9 Hz, 1H), 3.77 (q, *J* = 6.2 Hz, 1H), 3.67 (ddd, *J* = 11.5, 5.3, 2.2 Hz, 1H), 3.54 (s, 1H), 3.42–3.47 (m, 1H), 3.32–3.22 (m, 2H), 3.08 (dd, *J* = 12.9, 9.4 Hz, 1H), 3.01 (td, *J* = 6.5, 3.0 Hz, 1H), 2.84 (dd, *J* = 12.8, 6.2 Hz, 1H), 2.66 (s, 3H), 2.58 (dt, *J* = 6.9, 4.0 Hz, 1H), 1.95–1.75 (m, 2H), 1.61–1.47 (m, 3H), 1.44 (d, *J* = 17.1 Hz, 1H), 1.34 (d, *J* = 6.8 Hz, 1H), 1.32–1.24 (m, 2H), 1.23 (s, 1H), 1.22–1.11 (m, 1H), 1.06 (d, *J* = 6.1 Hz, 3H), 0.98 (s, 1H), 0.97–0.89 (m, 1H), 0.84 (d, *J* = 6.8 Hz, 3H), 0.79 (d, *J* = 6.4 Hz, 3H), 0.71 (d, *J* = 6.5 Hz, 3H), 0.71–0.62 (m, 4H). **^13^C NMR** (150 MHz, DMSO) δ 173.5, 173.0, 168.6, 137.7, 129.1, 128.2, 126.4, 97.5, 77.4, 76.0, 73.9, 72.4, 71.3, 67.3, 67.0, 61.5, 59.8, 49.6, 46.3, 36.9, 35.8, 33.5, 31.7, 31.5, 29.8, 29.8, 26.1, 25.3, 23.3, 19.2, 15.6, 15.3, 9.7, 7.5. **HRMS (ESI)** calculated for C_36_H_59_N_2_O_11_^+^ [M + H]^+^ 695.4119 found 695.4121.

#### 3.2.18. Colletopeptide A

To a solution of **4** (12 mg, 0.019 mmol, 1.0 equiv.) in THF (3 mL, 0.006 M) at 0 °C, TBAF was added (0.23 mL, 0.23 mmol, 12.0 equiv., 1.0 M in THF). The reaction mixture was warmed up to an ambient temperature, stirred for 6 h, and then quenched with a saturated aqueous solution of NH_4_Cl (3 mL). The aqueous layer was extracted with EtOAc (2 × 20 mL). The combined organic layers were washed with H_2_O (10 mL) and brine (10 mL), dried over anhydrous Na_2_SO_4_, and then filtered and concentrated in vacuo. Purification of the crude product was performed via flash chromatography on silica (hexanes/EtOAc = 4/1) to afford colletopeptide A (7 mg, 67%) as a colorless amorphous solid. **TLC**: Rf = 0.3 (hexanes/EtOAc = 3/1), UV & PMA stain. ${\left[\alpha \right]}_{D}^{20}$= −62.03 (c 0.8, MeOH). **^1^H NMR** (500 MHz, MeOD) δ 7.35–7.21 (m, 4H), 7.25–7.15 (m, 1H), 5.26 (dd, *J* = 10.7, 5.3 Hz, 1H), 4.85 (s, 1H), 4.76 (ddd, *J* = 10.6, 4.3, 2.2 Hz, 1H), 4.55 (s, 1H), 4.04 (dd, *J* = 8.3, 3.6 Hz, 1H), 3.78–3.57 (m, 1H), 3.22 (dd, *J* = 12.8, 10.7 Hz, 1H), 2.91 (dd, *J* = 12.8, 5.3 Hz, 1H), 2.71 (s, 3H), 2.70–2.60 (m, 1H), 2.05–1.92 (m, 1H), 1.94–1.83 (m, 1H), 1.69 (ddd, *J* = 15.8, 8.4, 2.2 Hz, 1H), 1.67–1.60 (m, 1H), 1.63–1.54 (m, 1H), 1.52–1.42 (m, 1H), 1.42–1.34 (m, 3H), 1.36–1.25 (m, 2H), 1.14 (d, *J* = 6.1 Hz, 3H), 1.10–1.01 (m, 1H), 1.01 (d, *J* = 6.9 Hz, 3H), 0.98–0.92 (m, 1H), 0.86 (d, *J* = 6.5 Hz, 3H), 0.80 (d, *J* = 6.6 Hz, 3H), 0.79–0.69 (m, 1H), 0.74 (d, *J* = 3.5 Hz, 3H). **^13^C NMR** (100 MHz, MeOD) δ 176.4, 175.2, 170.1, 138.4, 130.3, 129.6, 127.9, 77.9, 69.1, 68.6, 62.1, 51.8, 48.7, 40.2, 37.4, 35.3, 33.3, 32.7, 31.7, 30.9, 27.6, 27.1, 24.9, 23.5, 16.3, 15.9, 10.3, 7.8. **HRMS (ESI)** calculated for C_30_H_49_N_2_O_6_^+^ [M + H]^+^ 533.3591 found 533.3578.

## 4. Conclusions

In conclusion, we have developed a concise and stereoselective synthesis of colletopeptide A (**1**) and colletotrichamide A (**2**) from commercially available alcohol **13** (with the longest linear sequences at 15 and 17 steps, separately), which served to support the structural elucidation of these natural products. The cyclic tridepsipeptide based aglycone **4** was synthesized enantioselectively in 14 steps with a high efficiency and overall yield, which has the potential to serve as an advanced intermediate for the synthesis of other family members, such as colletotrichamide B–D. The application of our synthetic strategy to the total synthesis other cyclic depsipeptides will be reported in due course.

## Data Availability

Copies of the ^1^H and ^13^C NMR spectra, as well as associated discussion and structural assignments, are provided in the Supporting Information.

## Supplementary Materials

The following supporting information can be downloaded at: https://www.mdpi.com/article/10.3390/molecules28207194/s1, Figures S1–S36: Copies of NMR spectra (^1^H and ^13^C) of **1**–**5**, **9**, **10**, **12**, **16**, and **18**–**26**.
