# Supplementary material for: Total Syntheses of Colletopeptide A and Colletotrichamide A
Source: Molecules. 2023 Oct 20;28(20):7194. doi: 10.3390/molecules28207194 (PMC10608858; doi:10.3390/molecules28207194)

Supplementary Information

Table of Contents

1. NMR comparison of natural and synthetic colletotrichamide A and colletopepetide A
2. NMR spectra

1. NMR comparison of natural and synthetic colletotrichamide A and colletopepetide A

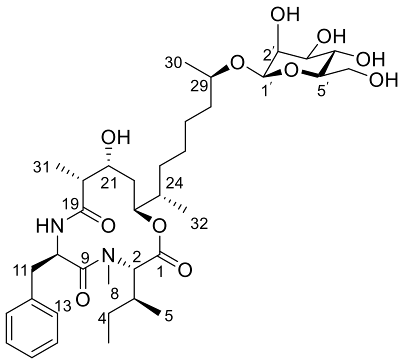

Colletotrichamide A

No.	Colletotrichamide A		
	**Natural (δ_3)**	**Synthetic (δ_4)**	**Δ_δ=δ_3-δ_4**
**1**	168.6	168.6	0.0
**2**	59.7	59.8	-0.1
**3**	29.8	29.8	0.0
**4**	23.3	23.3	0.0
**5**	9.8	9.7	0.1
**6**	15.3	15.3	0.0
**7**			
**8**	29.8	29.8	0.0
**9**	173.0	173.0	0.0
**10**	49.6	49.6	0.0
**11**	35.8	35.8	0.0
**12**	137.7	137.7	0.0
**13**	129.1	129.1	0.0
**14**	128.2	128.2	0.0
**15**	126.4	126.4	0.0
**16**	128.2	128.2	0.0
**17**	129.1	129.1	0.0
**18**			
**19**	173.5	173.5	0.0
**20**	46.3	46.3	0.0
**21**	67.0	67.0	0.0
**22**	31.4	31.5	-0.1
**23**	76.0	76.0	0.0
**24**	33.5	33.5	0.0
**25**	31.7	31.7	0.0
**26**	26.1	26.1	0.0
**27**	25.3	25.3	0.0
**28**	36.9	36.9	0.0
**29**	72.3	72.4	-0.1

**30**	19.2	19.2	0.0
**31**	7.5	7.5	0.0
**32**	15.6	15.6	0.0
**1′**	97.5	97.5	0.0
**2′**	71.2	71.3	-0.1
**3′**	73.9	73.9	0.0
**4′**	67.3	67.3	0.0
**5′**	77.4	77.4	0.0
**6′**	61.4	61.5	-0.1

**No.**	Colletotrichamide A		
	**Natural (δ_1) δH (mult., *J*, Hz)**	**Synthetic (δ_2) δH (mult., *J*, Hz)**	**$\Delta_\delta=\delta_1-\delta_2$ δH (mult., *J*, Hz)**
**1**			
**2**	4.77(d, 11.5)	4.77(d, 11.6)	0.00
**3**	1.83 (m)	1.86 (m)	-0.03
**4**	0.95 (m)<br>0.68 (m)	0.95 (m)<br>0.67 (m)	0.00<br>0.01
**5**	0.68(m)	0.67 (m)	0.01
**6**	0.79 (d, 6.5)	0.79 (d, 6.4)	0.00
**7**			
**8**	2.66 (s)	2.66 (s)	0.00
**9**			
**10**	5.06 (dt, 9.5, 6.0)	5.06 (dt, J = 9.4, 6.2)	0.00
**11**	3.08 (dd, 13.0, 9.5)<br>2.84 (dd, 13.0, 6.0)	3.08 (dd, 12.9, 9.4)<br>2.84 (dd, 12.8, 6.2)	0.00<br>0.00
**12**			
**13**	7.22 (d, 7.0)	7.23 (d, 4.0)	-0.01
**14**	7.24 (d, 7.0)	7.26 (d, 7.1)	-0.02
**15**	7.19 (m)	7.19 (m)	0.00
**16**	7.24 (d, 7.0)	7.26 (d, 7.1)	-0.02
**17**	7.22 (d, 7.0)	7.23 (d, 4.0)	-0.01
**18**	8.61 (d, 9.5)	8.58 (d, 9.5)	0.03
**19**			
**20**	2.57 (dq, 6.5, 3.5)	2.58 (dt, 6.9, 4.0),	-0.01
**21**	3.87 (ddd, 8.5, 5.5, 3.5)	3.88 (d, 8.9)	-0.01
**21-OH**	4.51 (d, 5.5)	4.50 (d, 5.1)	0.01
**22**	1.51 (m)<br>1.44 (dd, 15.0, 3.5)	1.51 (m)<br>1.44 (d, 17.1)	0.00<br>0.00
**23**	4.63 (dt, 10.5, 3.0)	4.64 (d, 10.0),	-0.01
**24**	1.86 (m)	1.86 (m)	0.00
**25**	1.55 (m)	1.51 (m)	0.04

	0.99 (dd, 10.5, 3.0)	0.98 (s)	0.01
**26**	1.23 (m)<br>1.17 (m)	1.23 (s)<br>1.16 (m)	0.00<br>0.01
**27**	1.26 (m)	1.26 (m)	0.00
**28**	1.51 (m)<br>1.34 (dt, 13.5, 6.5)	1.51 (m)<br>1.34 (d, 6.8)	0.00<br>0.00
**29**	3.77(sextet, 6.0)	3.77 (q, 6.2)	0.00
**30**	1.05 (d, 6.0)	1.06 (d, 6.1)	-0.01
**31**	0.84 (d, 6.5)	0.84 (d, 6.8)	0.00
**32**	0.71 (d, 6.5)	0.71 (d, 6.5)	0.00
**1′**	4.43 (s)	4.43 (s)	0.00
**2′**	3.54 (dd, 5.0, 2.0)	3.54 (s)	0.00
**2′-OH**	4.18 (d, 5.0)	4.17 (d, 4.3)	0.01
**3′**	3.26 (m)	3.26 (m)	0.00
**3′-OH**	4.53 (d, 5.0)	4.53 (s)	0.00
**4′**	3.26 (m)	3.26 (m)	0.00
**4′-OH**	4.71 (d, 4.5)	4.71 (s)	0.00
**5′**	3.01 (ddd, 8.5, 5.5, 2.0)	3.01 (dt, 6.5, 3.0)	0.00
**6′**	3.67 (ddd, 11.5, 5.5, 2.0)<br>3.44 (dt, 11.5, 5.5)	3.67 (ddd, 11.5, 5.3, 2.2)<br>3.45 (m)	0.00<br>-0.01
**6′-OH**	4.37 (t, 5.5)	4.35 (t, 5.5)	0.02

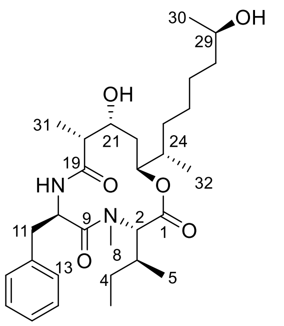

Colletopeptide A

No.	Colletopeptide A		
	**Natural (δ_3)**	**Synthetic (δ_4)**	**Δ_δ=δ_3-δ_4**
**1**	170.1	170.1	0.0
**2**	62.1	62.1	0.0
**3**	31.7	31.7	0.0
**4**	24.9	24.9	0.0
**5**	10.3	10.3	0.0
**6**	15.9	15.9	0.0
**7**			
**8**	30.8	30.9	-0.1
**9**	175.2	175.2	0.0
**10**	51.8	51.8	0.0
**11**	37.4	37.4	0.0
**12**	138.4	138.4	0.0
**13**	130.3	130.3	0.0
**14**	129.6	129.6	0.0
**15**	127.9	127.9	0.0
**16**	129.6	129.6	0.0
**17**	130.3	130.3	0.0
**18**			
**19**	176.4	176.4	0.0
**20**	48.7	48.7	0.0
**21**	69.1	69.1	0.0
**22**	32.7	32.7	0.0
**23**	77.8	77.9	-0.1
**24**	35.3	35.3	0.0
**25**	33.3	33.3	0.0
**26**	27.6	27.6	0.0
**27**	27.1	27.1	0.0
**28**	40.2	40.2	0.0
**29**	68.5	68.6	-0.1
**30**	23.5	23.5	0.0
**31**	7.8	7.8	0.0
**32**	16.3	16.3	0.0

No.	Colletopeptide A		
	**Natural (δ_1)**<br>**δH (mult., *J*, Hz)**	**Synthetic (δ_2)**<br>**δH (mult., *J*, Hz)**	**$\Delta_\delta=\delta_1-\delta_2$**<br>**δH (mult., *J*, Hz)**
**1**			
**2**	4.84 (d, 11.6)	4.85 (s)	-0.01
**3**	1.89 (m)	1.89 (m)	0.00
**4**	0.95 (m)<br>0.75 (m)	0.95 (m)<br>0.74 (m)	0.00<br>0.01
**5**	0.74 (d, 3.8)	0.74 (d, 3.5)	0.00
**6**	0.86 (d, 6.5)	0.86 (d, 6.5)	0.00
**7**			
**8**	2.71 (s)	2.71 (s)	0.00
**9**			
**10**	5.26 (dd, 10.8, 5.3)	5.26 (dd, J = 10.7, 5.3)	0.00
**11**	3.22 (dd, 12.8, 10.8)<br>2.91 (dd, 12.8, 5.3)	3.22 (dd, 12.8, 10.7)<br>2.91 (dd, 12.8, 5.3)	0.00<br>0.00
**12**			
**13**	7.26 (overlap)	7.26 (m)	0.00
**14**	7.26 (overlap)	7.26 (m)	0.00
**15**	7.21 (m)	7.21 (m)	0.00
**16**	7.26 (overlap)	7.26 (m)	0.00
**17**	7.26 (overlap)	7.26 (m)	0.00
**18**			
**19**			
**20**	2.65 (m)	2.66 (m)	-0.01
**21**	4.03 (dd, 8.4, 3.9)	4.04 (dd, 8.3, 3.6)	-0.01
**21-OH**		4.55 (s)	
**22**	1.69 (ddd, 15.7, 8.4, 2.0)<br>1.59 (dd, 15.7, 3.9)	1.69 (ddd, 15.8, 8.4, 2.2)<br>1.59 (m)	0.00<br>0.00
**23**	4.76 (d-like, 10.4)	4.76 (ddd, 10.6, 4.3, 2.2)	0.00
**24**	1.98 (m)	1.99 (m)	-0.01
**25**	1.62 (m)<br>1.05 (m)	1.63 (m)<br>1.05 (m)	-0.01<br>0.00
**26**	1.29 (m)	1.30 (m)	-0.01
**27**	1.36 (overlap)	1.37 (m)	-0.01
**28**	1.46 (m)<br>1.36 (overlap)	1.47 (m)<br>1.37 (m)	-0.01<br>-0.01
**29**	3.70 (m)	3.71 (m)	-0.01
**30**	1.14 (d, 6.2)	1.14 (d, 6.1)	0.00
**31**	1.00 (d, 6.8)	1.01 (d, 6.9)	-0.01
**32**	0.80 (d, 6.6)	0.80 (d, 6.6)	0.00

2. NMR spectra

Figures S1 ^{1}H NMR Spectrum of **9**

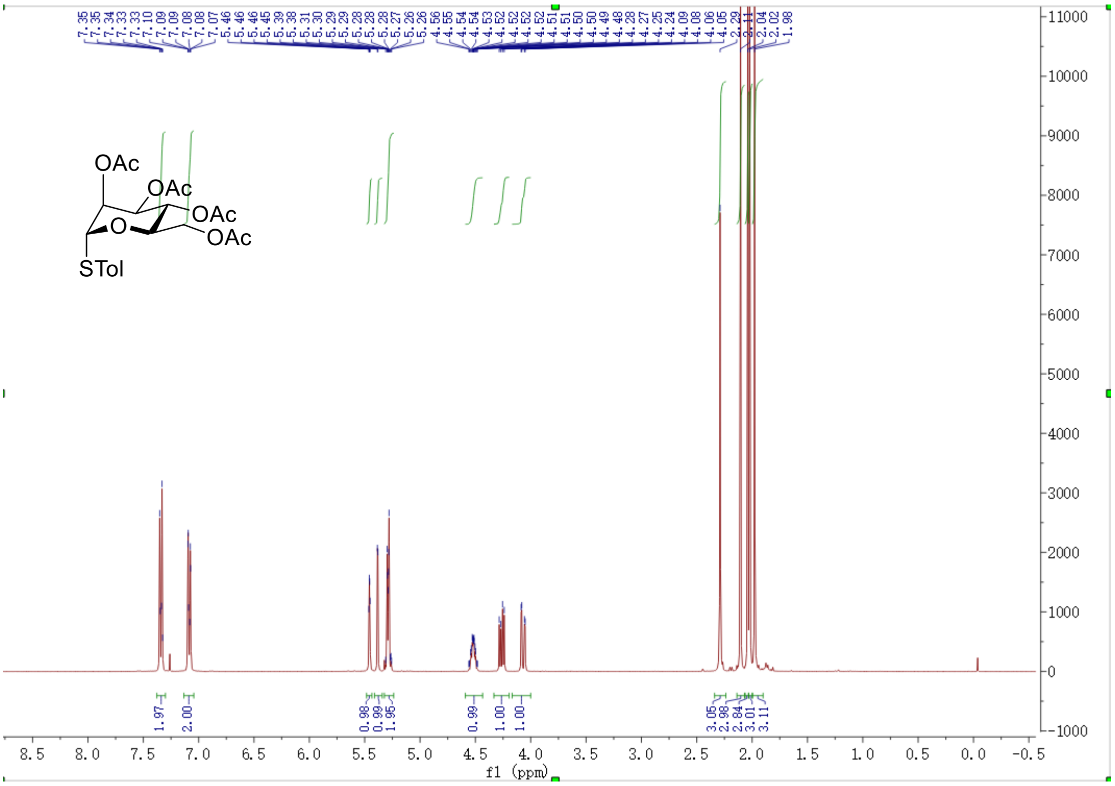

Figures S2 ^{13}C NMR Spectrum of **9**

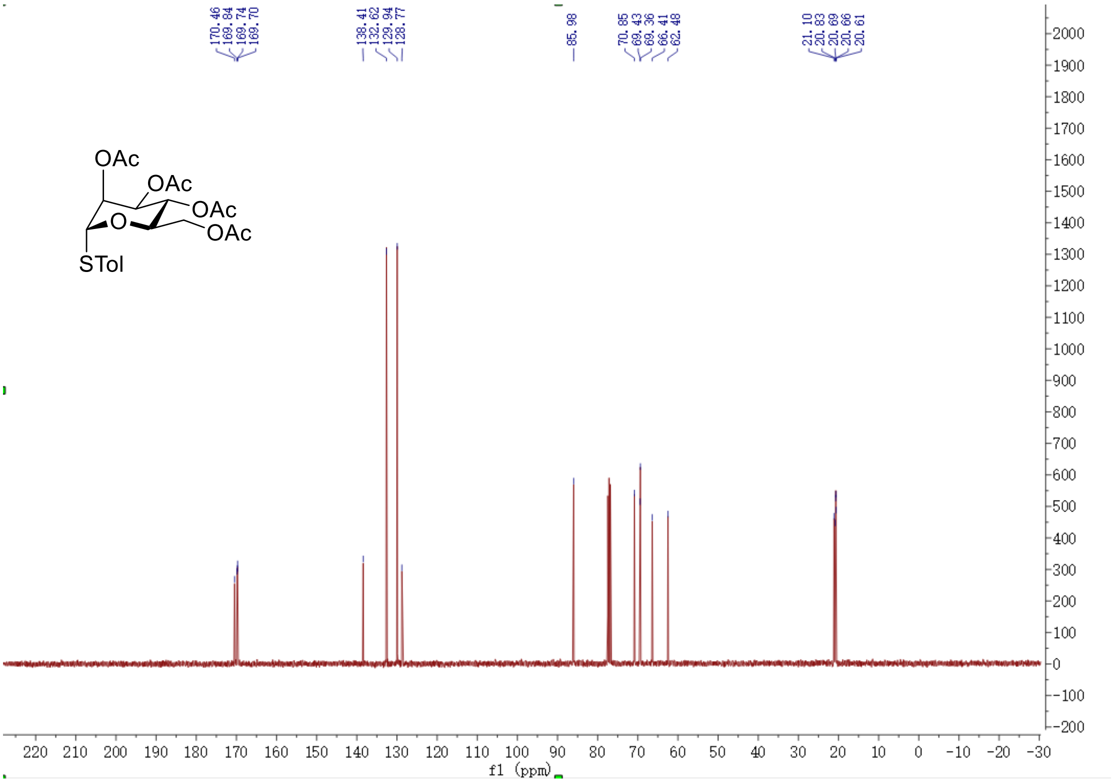

Figures S3 ^{1}H NMR Spectrum of **10**

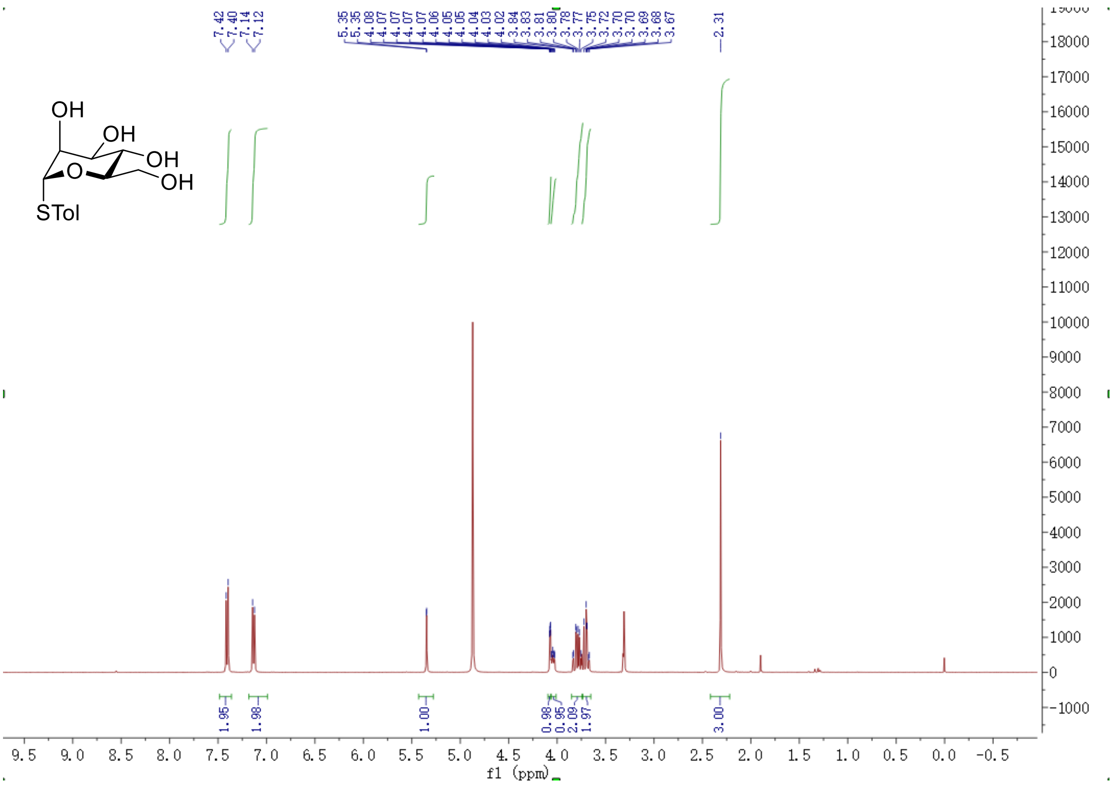

Figures S4 ^{13}C NMR Spectrum of **10**

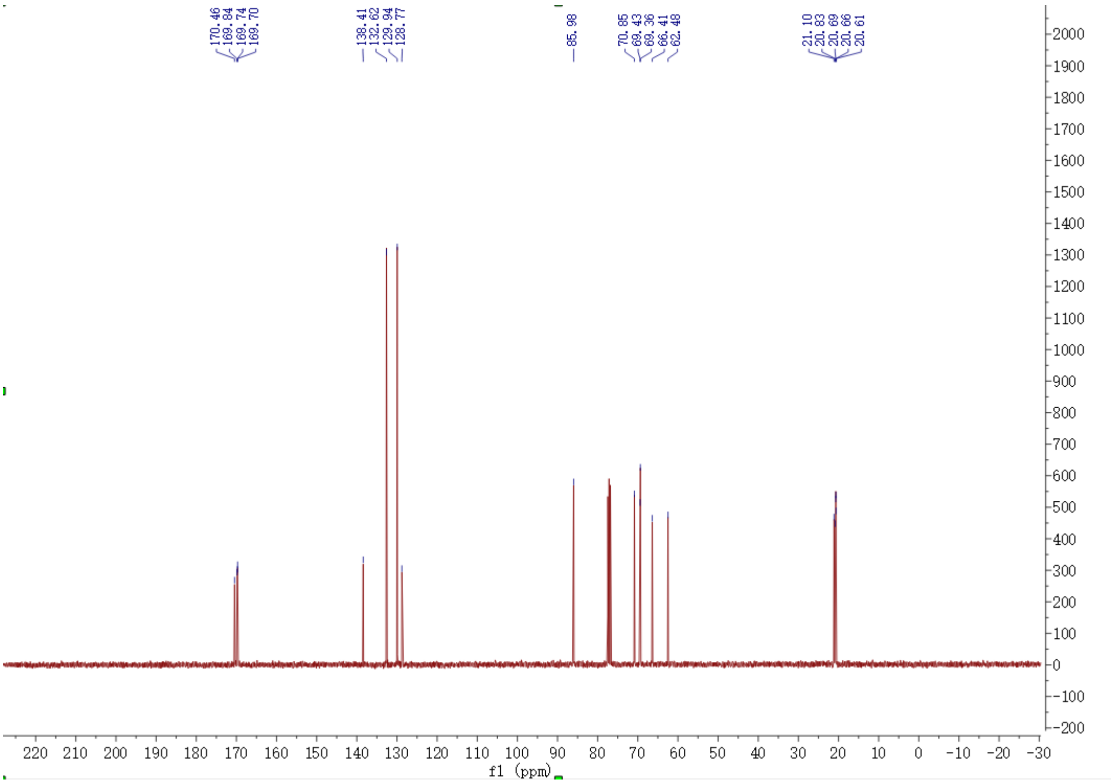

Figures S5 ^{1}H NMR Spectrum of **12**

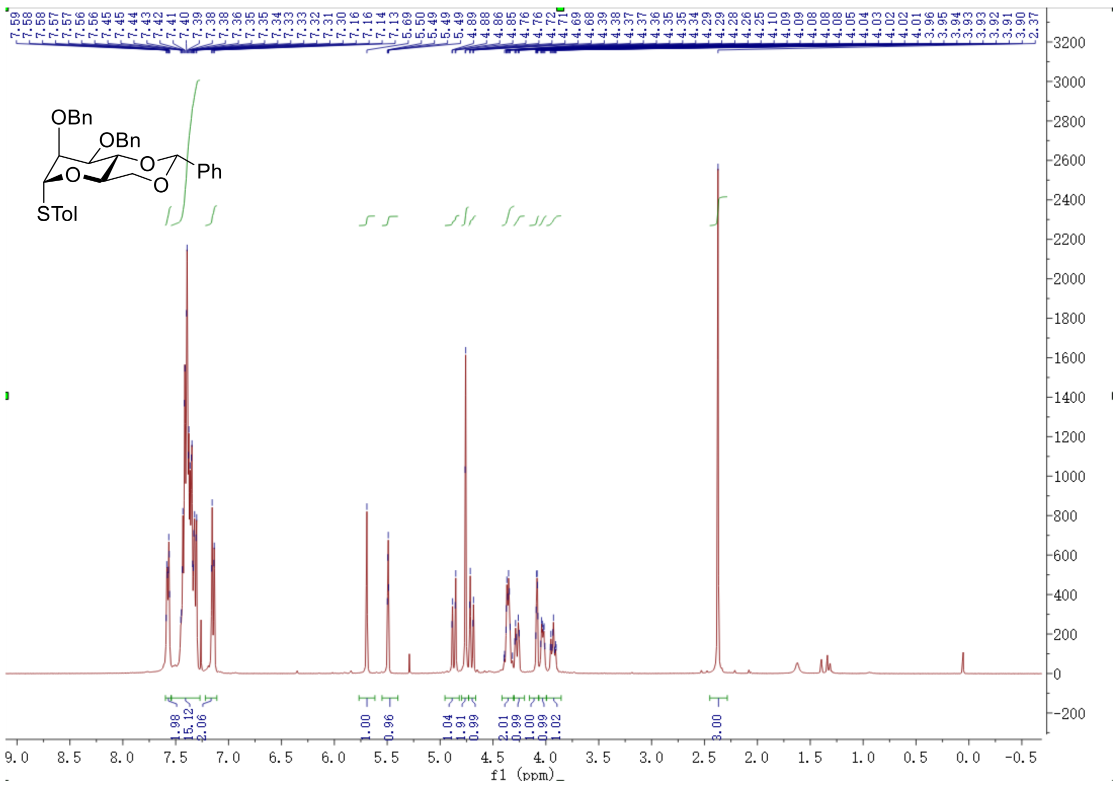

Figures S6 ^{13}C NMR Spectrum of **12**

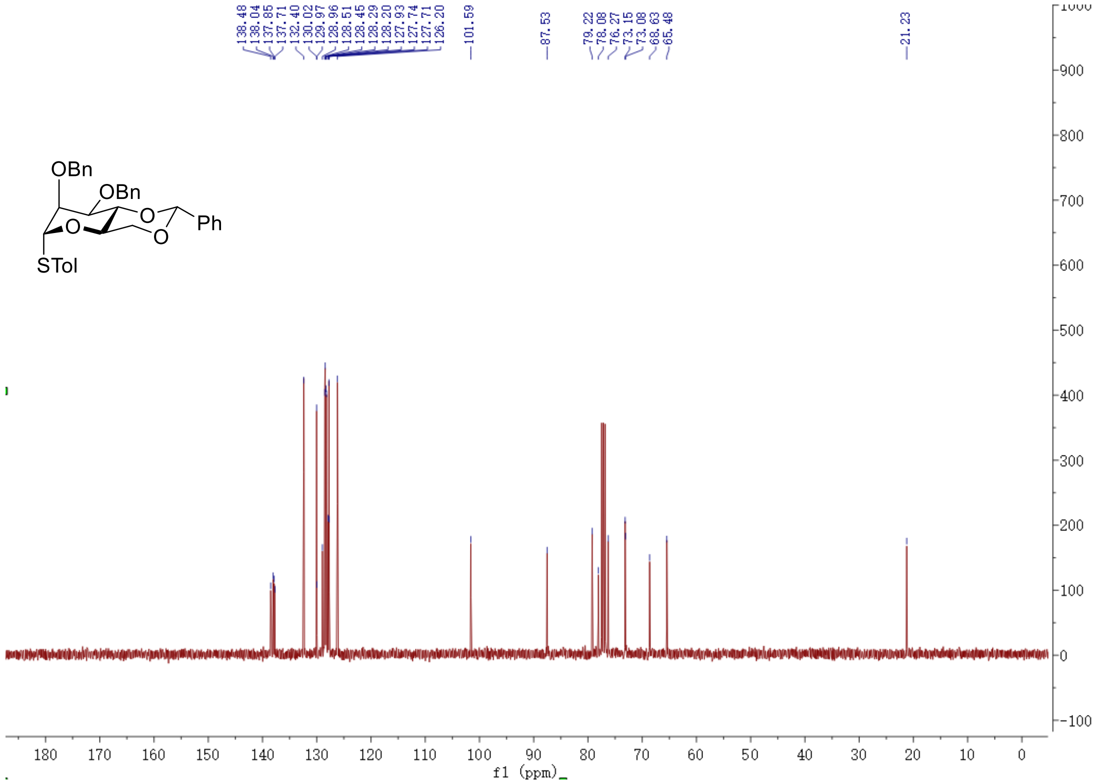

Figures S7 ¹H NMR Spectrum of **3**

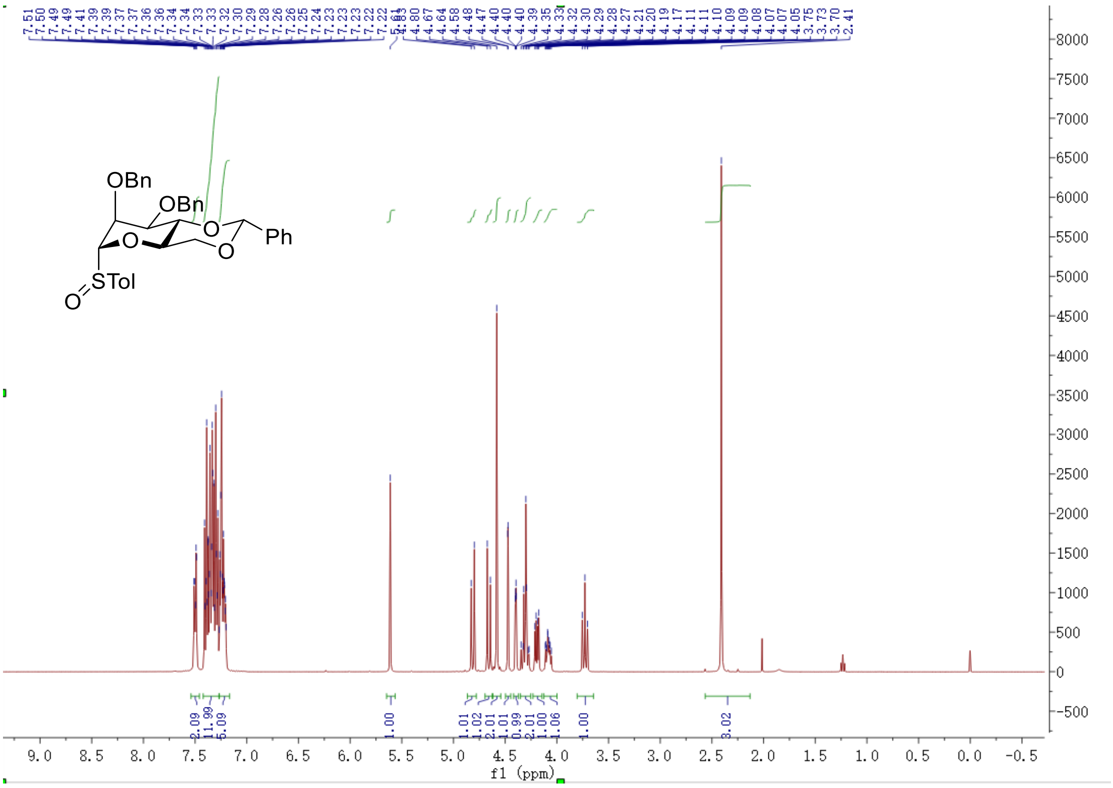

Figures S8 ¹³C NMR Spectrum of **3**

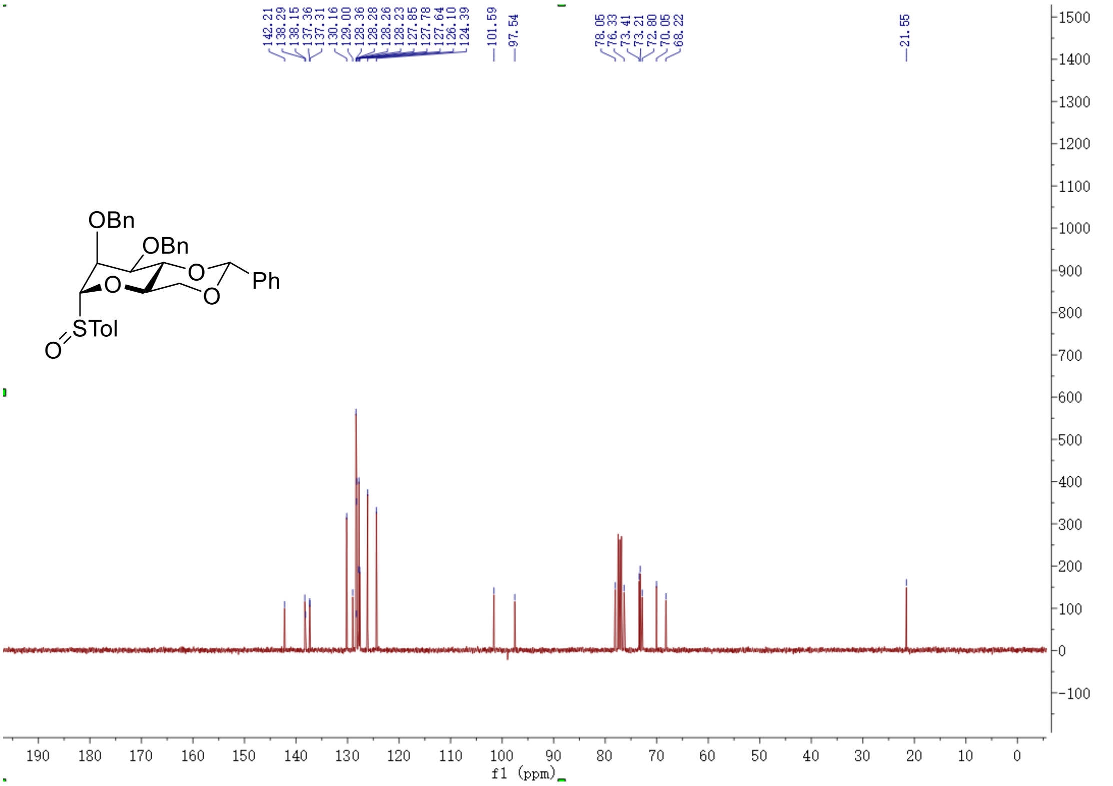

Figures S9 ^{1}H NMR Spectrum of **5**

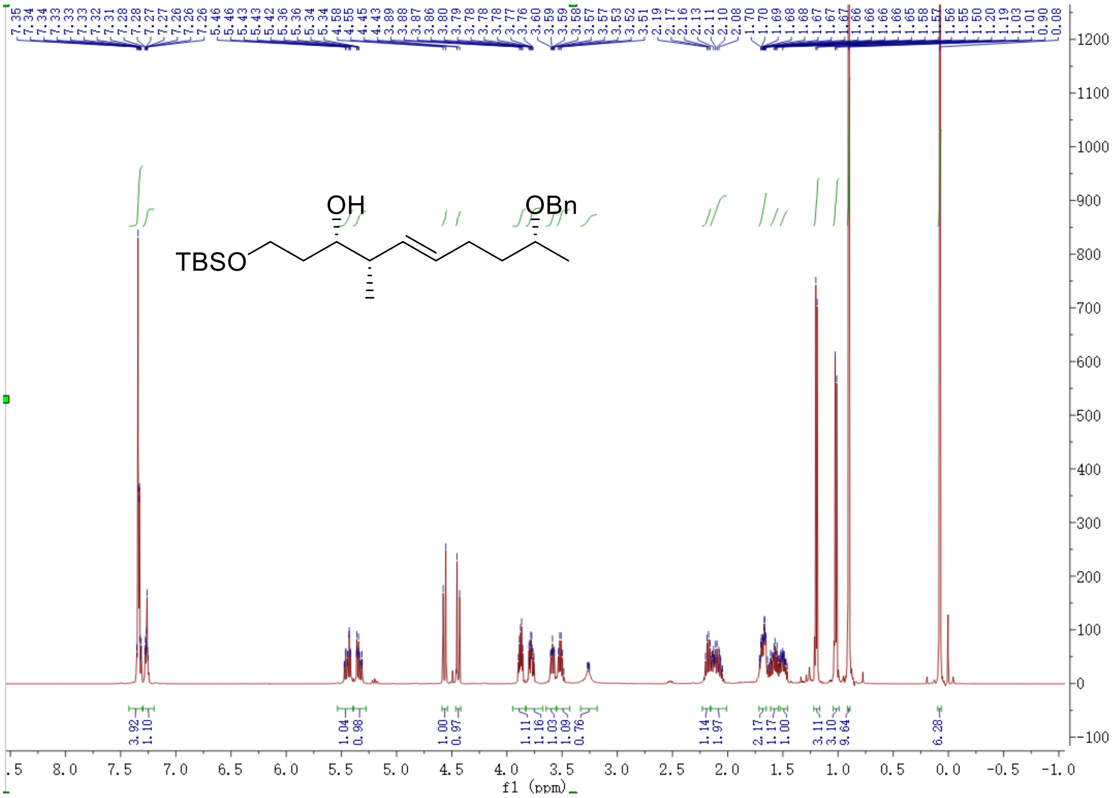

Figures S10 ^{13}C NMR Spectrum of **5**

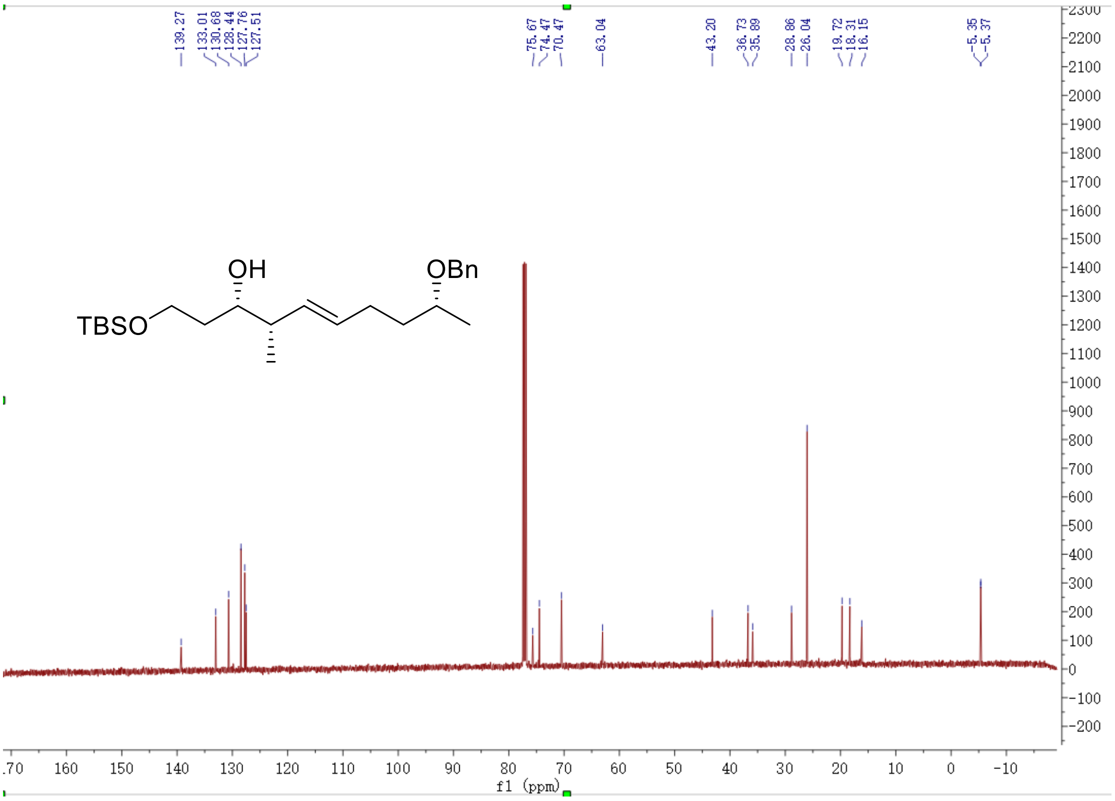

Figures S11 ^{1}H NMR Spectrum of **16**

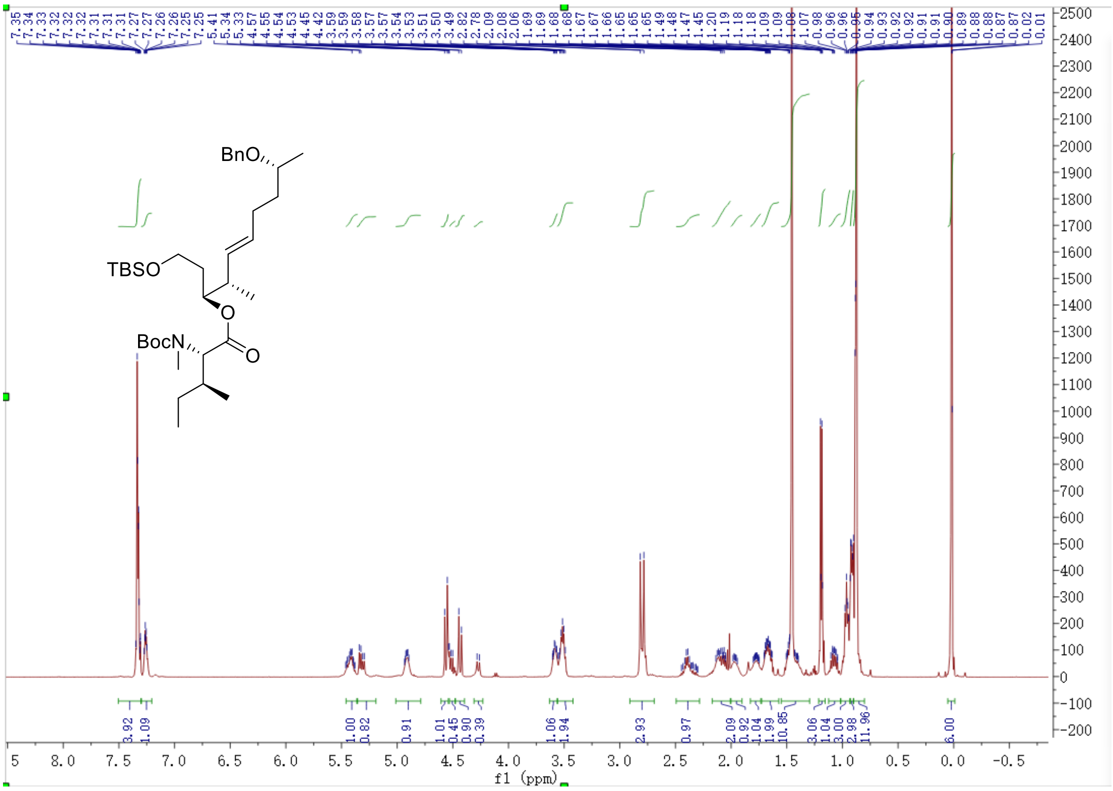

Figures S12 ^{13}C NMR Spectrum of **16**

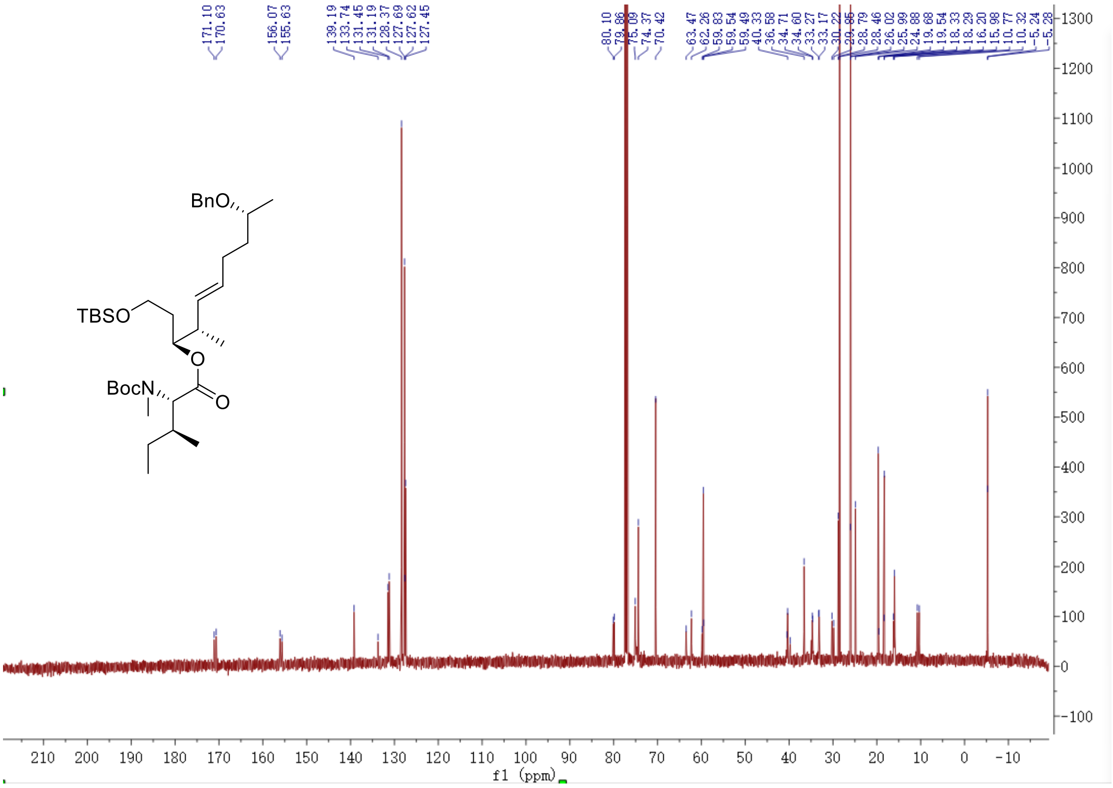

Figures S13 ^{1}H NMR Spectrum of **18**

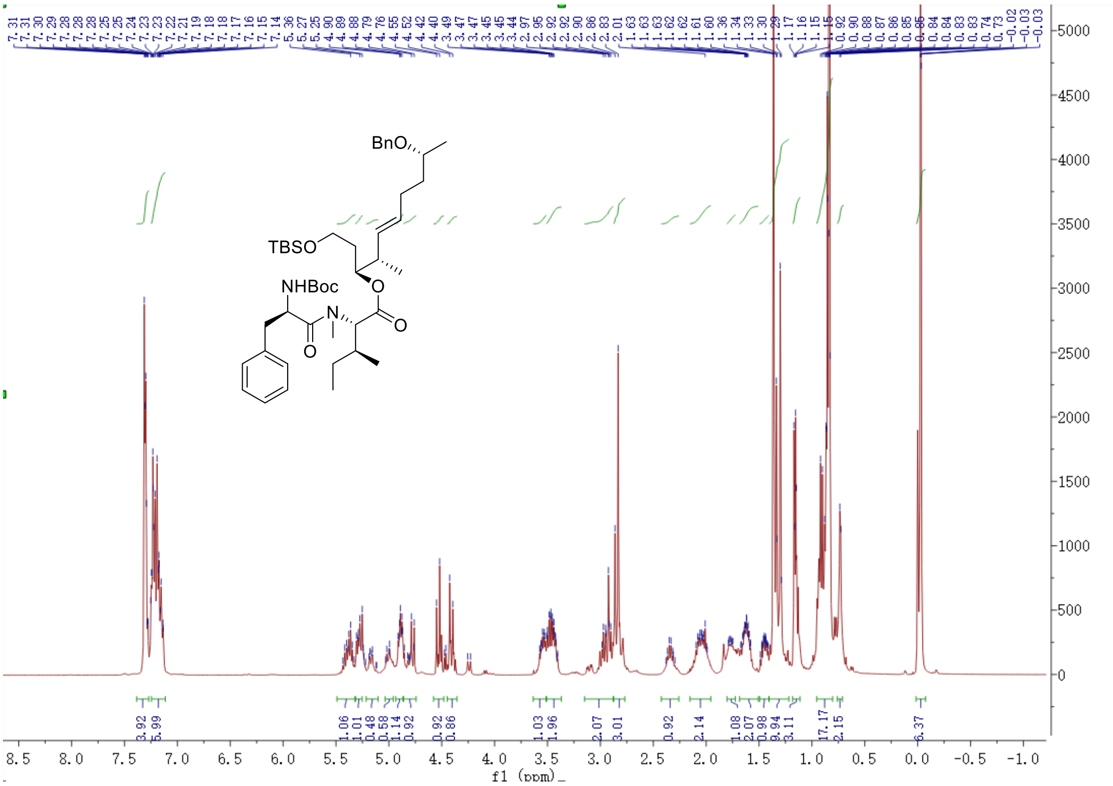

Figures S14 ^{13}C NMR Spectrum of **18**

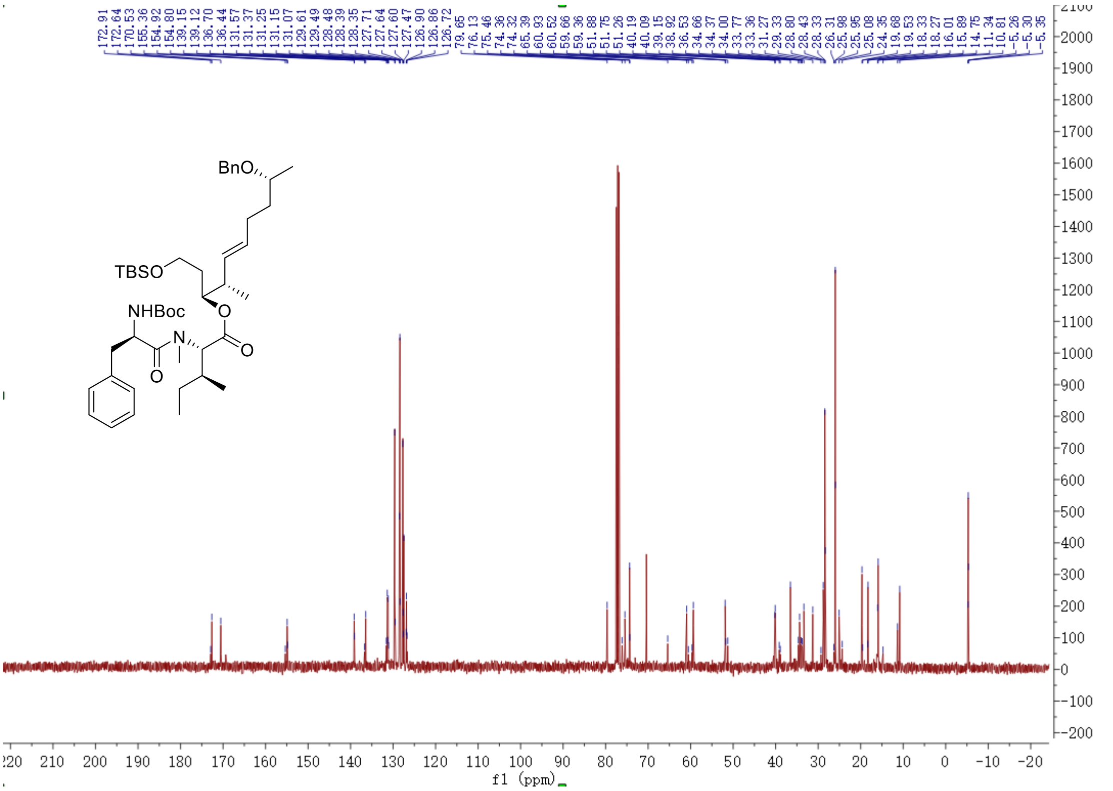

Figures S15 ^{1}H NMR Spectrum of **19**

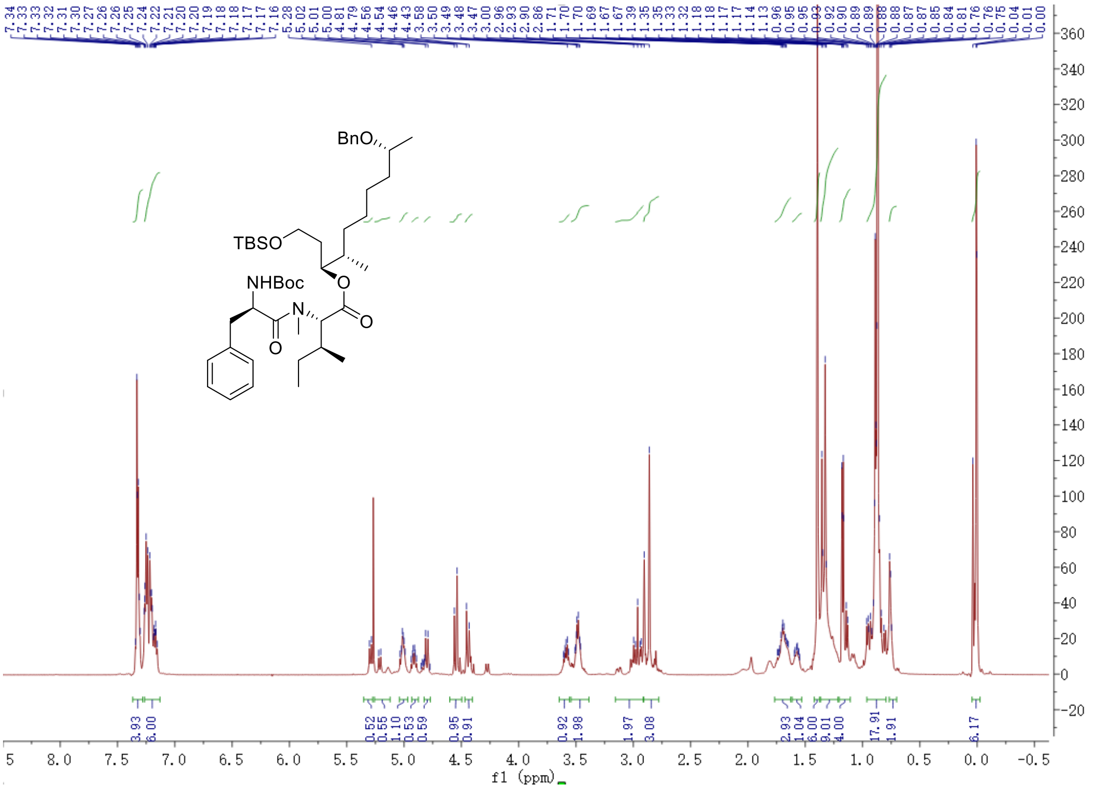

Figures S16 ^{13}C NMR Spectrum of **19**

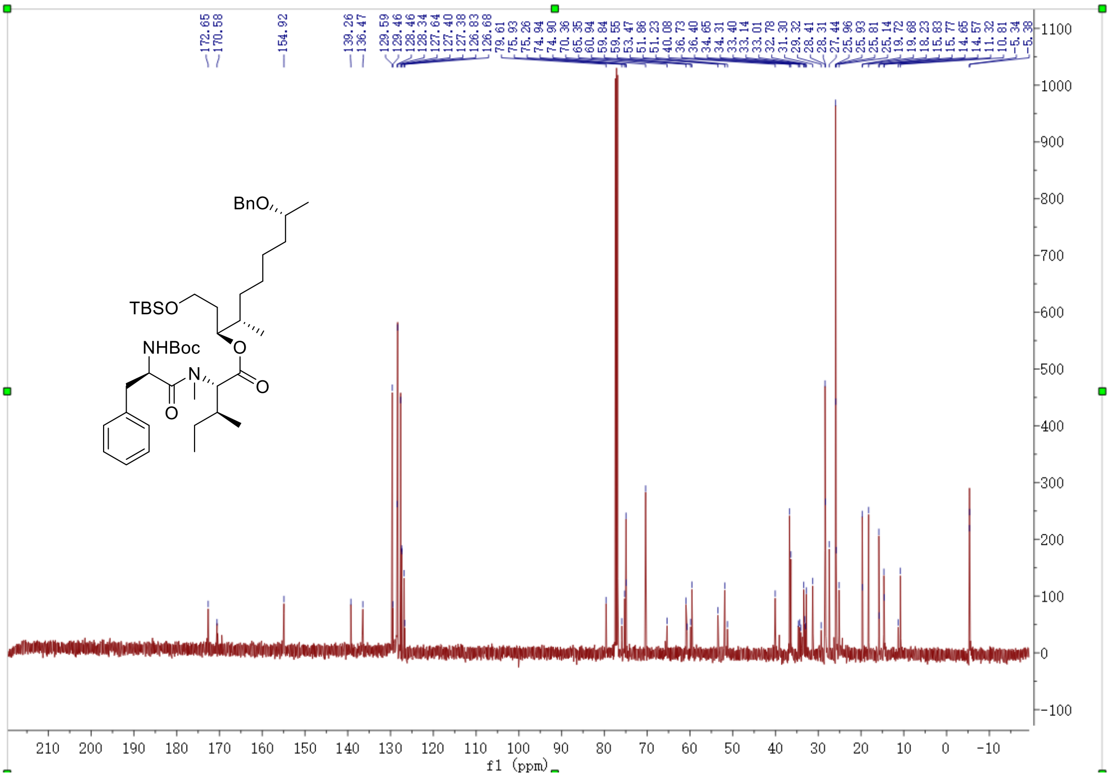

Figures S17 ^{1}H NMR Spectrum of **20**

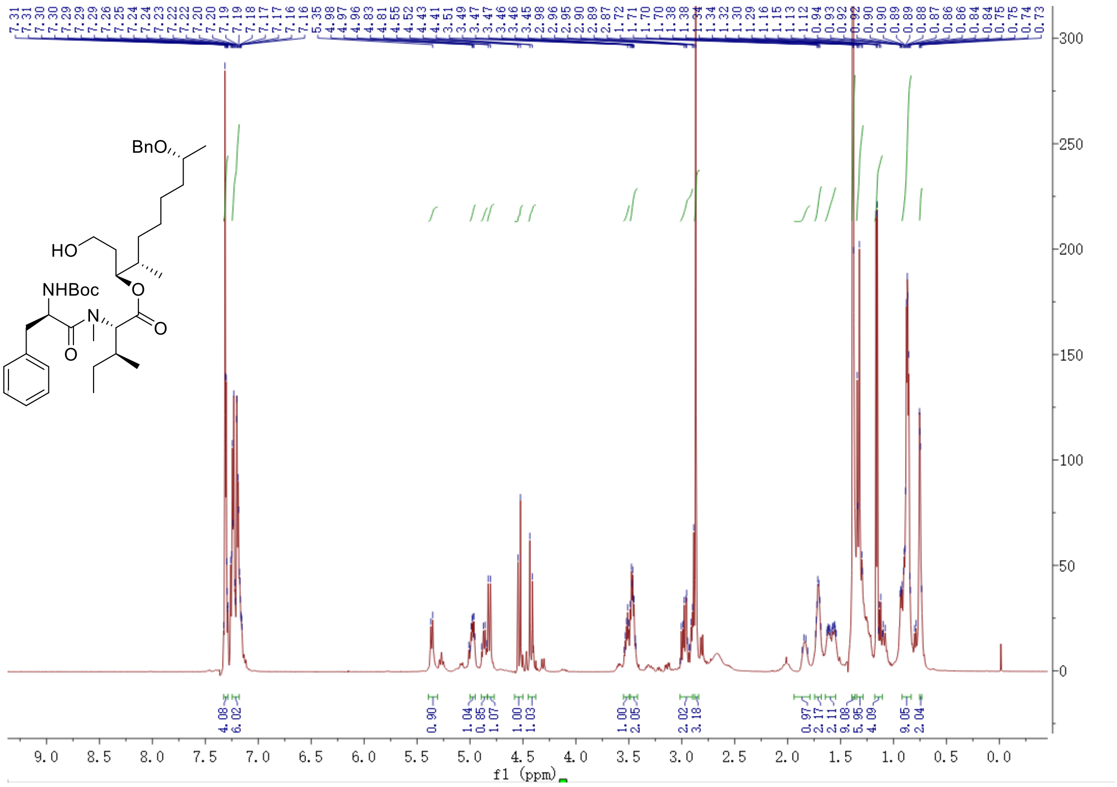

Figures S18 ^{13}C NMR Spectrum of **20**

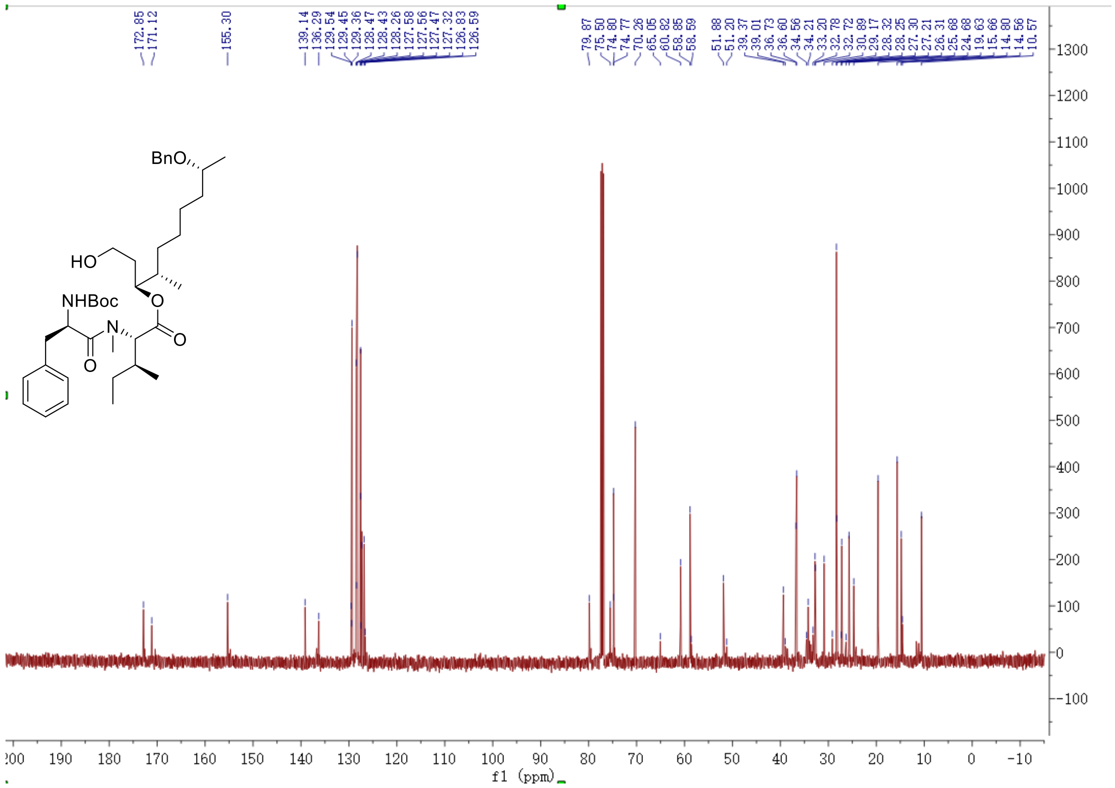

Figures S19 ^{1}H NMR Spectrum of **21**

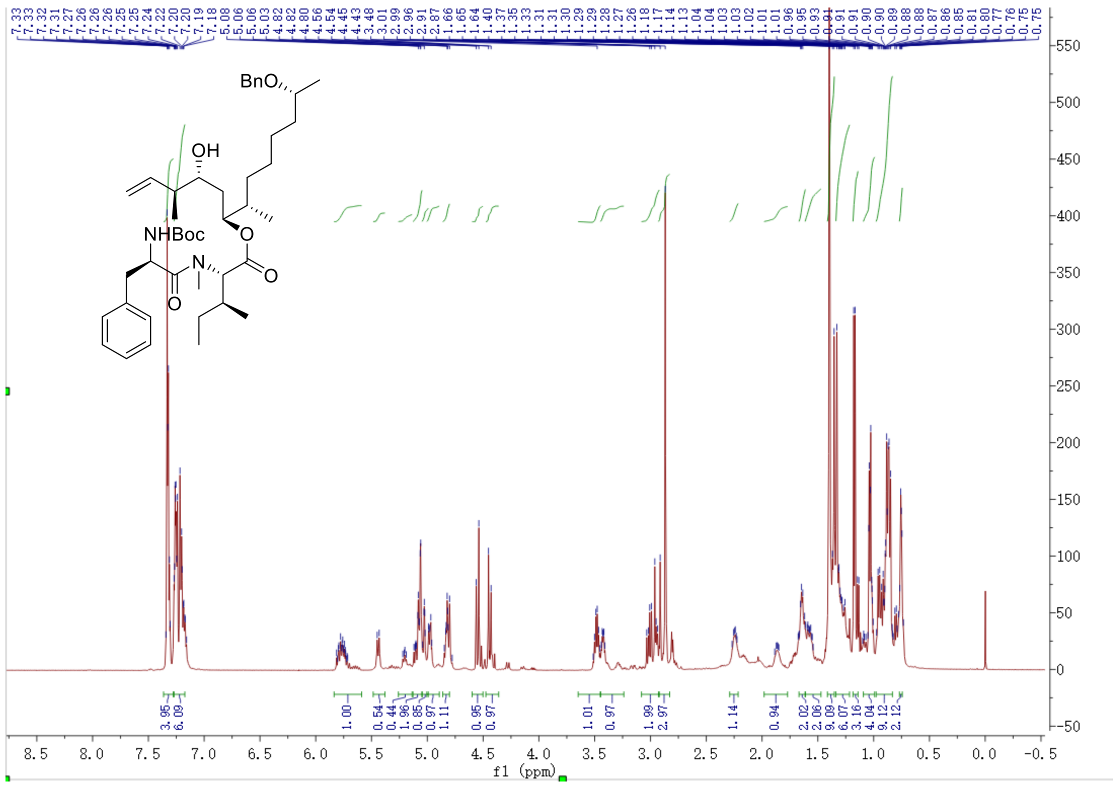

Figures S20 ^{13}C NMR Spectrum of **21**

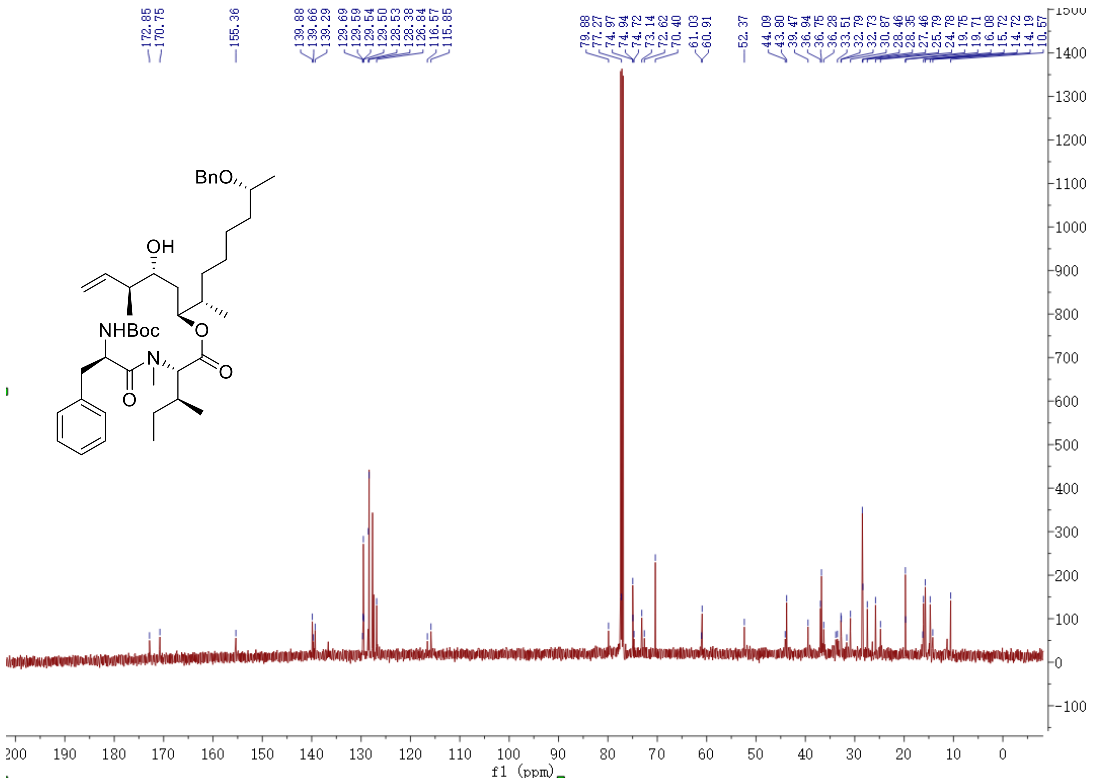

Figures S21 ^{1}H NMR Spectrum of **22**

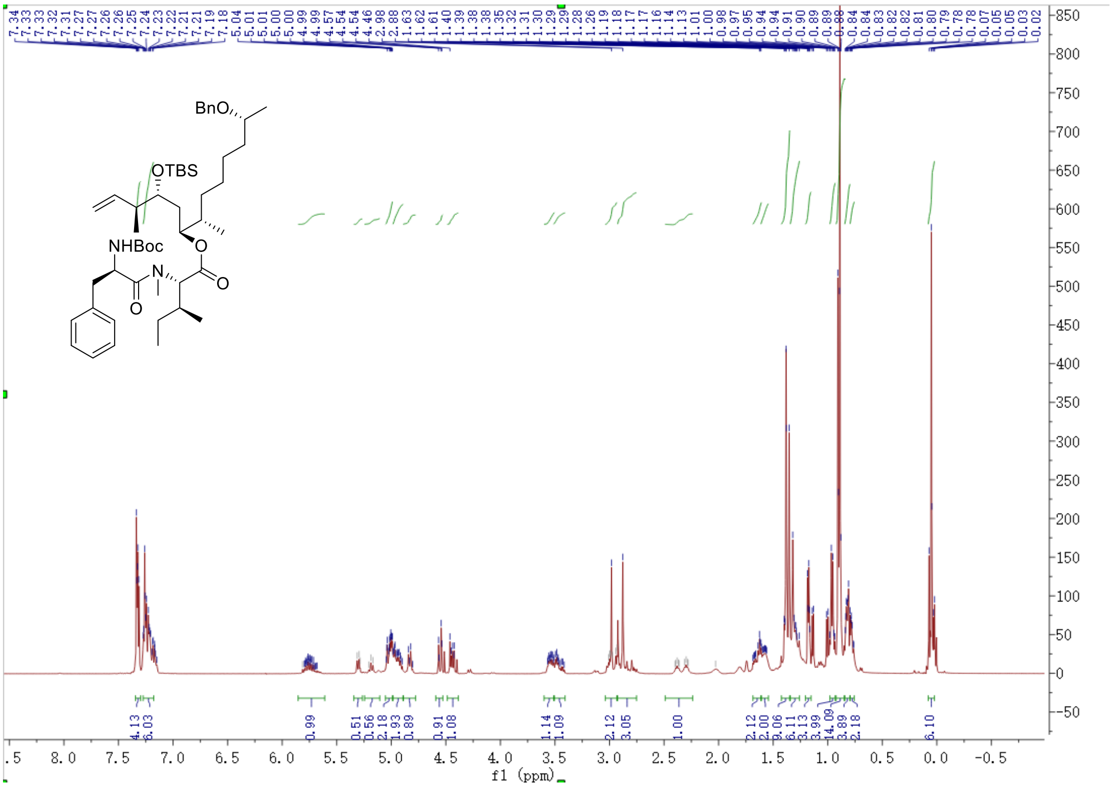

Figures S22 ^{13}C NMR Spectrum of **22**

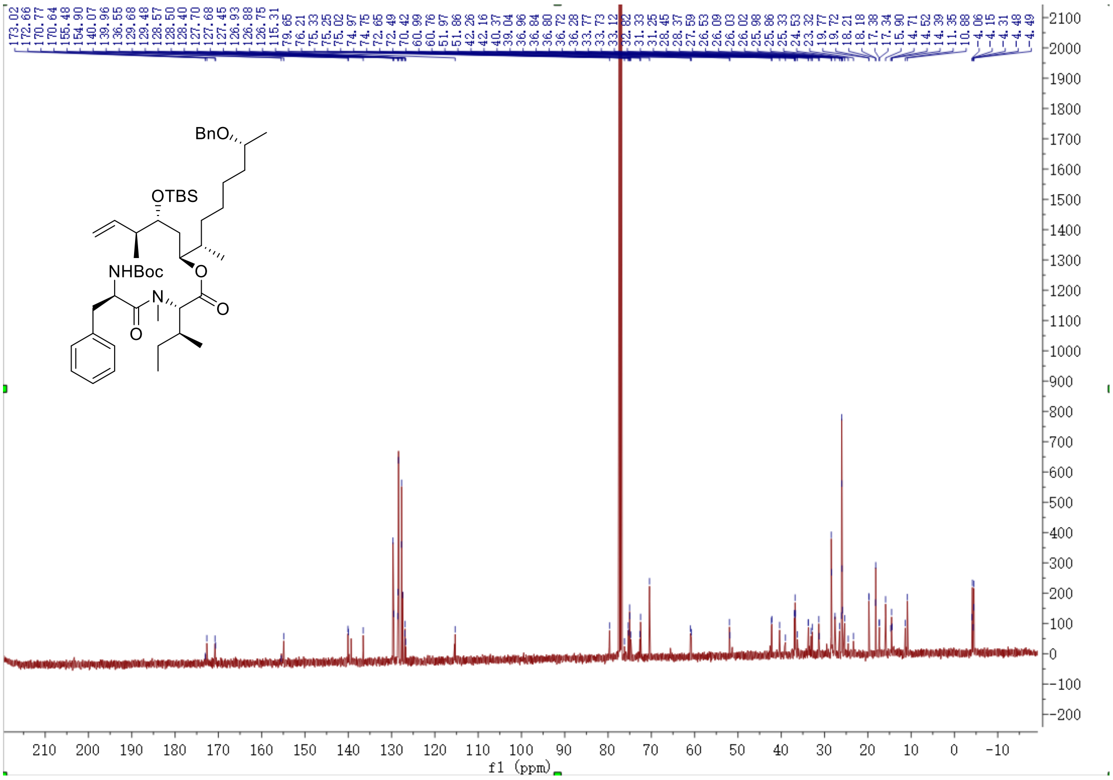

Figures S23 ^{1}H NMR Spectrum of **23**

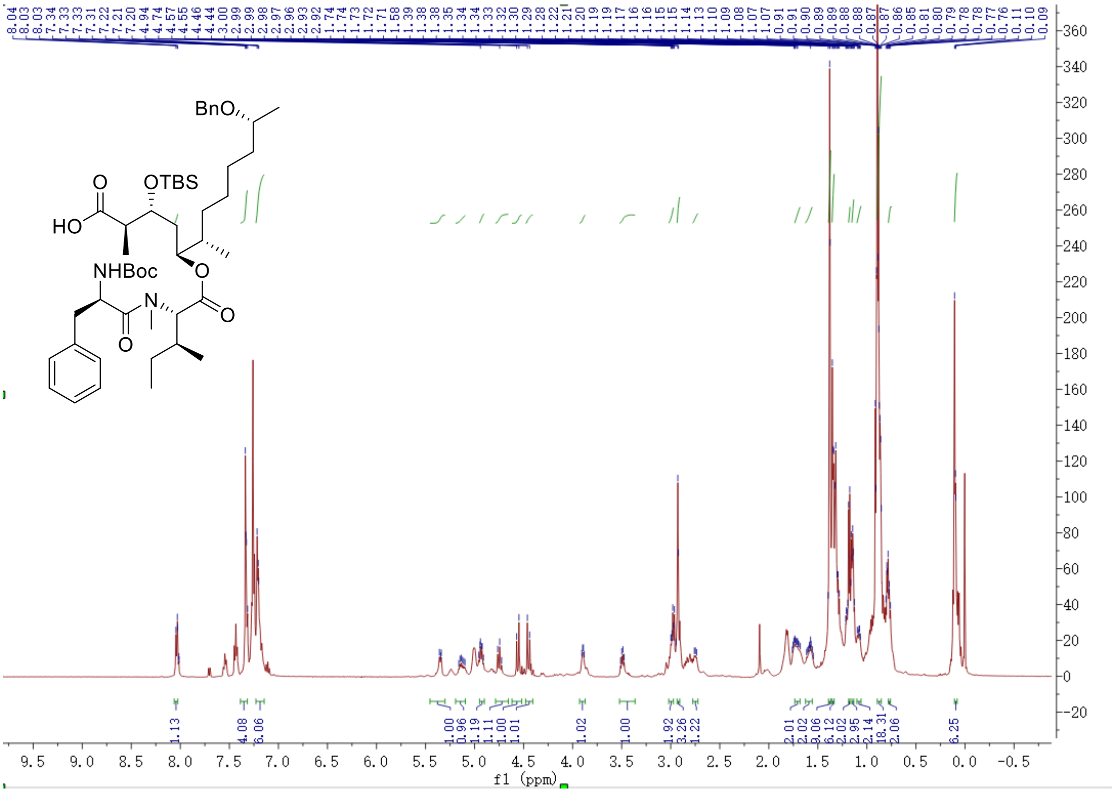

Figures S24 ^{13}C NMR Spectrum of **23**

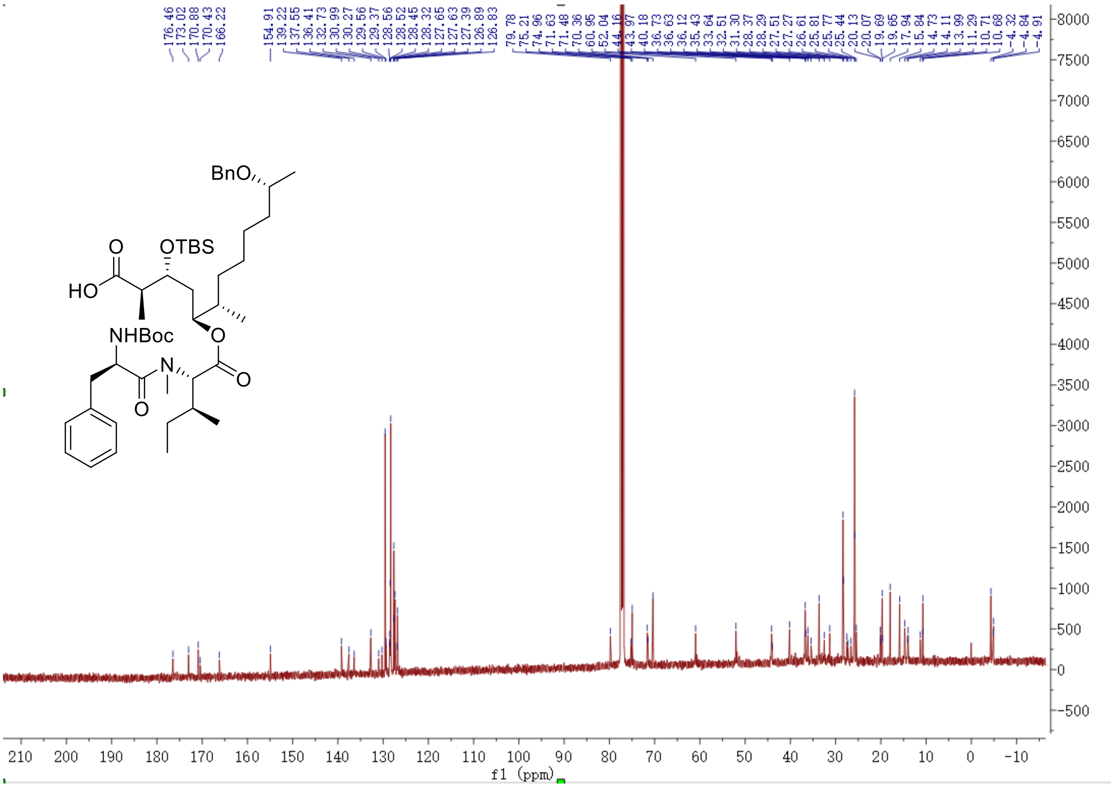

Figures S25 ^{1}H NMR Spectrum of **24**

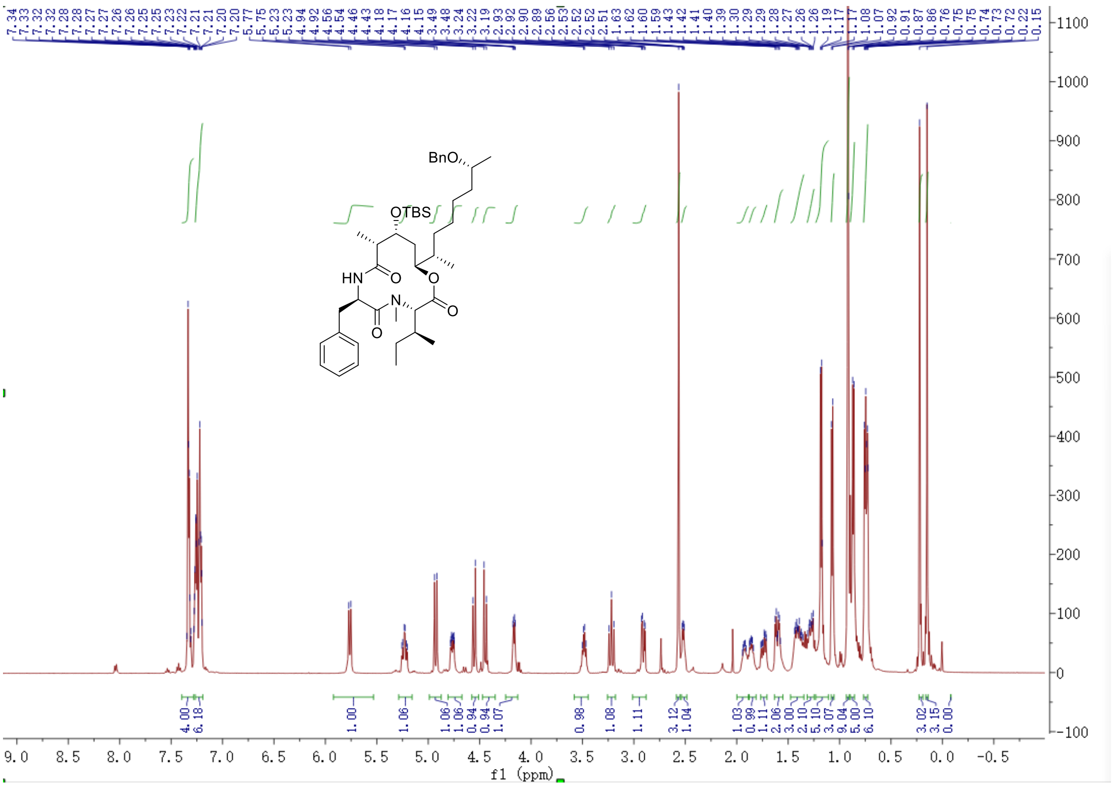

Figures S26 ^{13}C NMR Spectrum of **24**

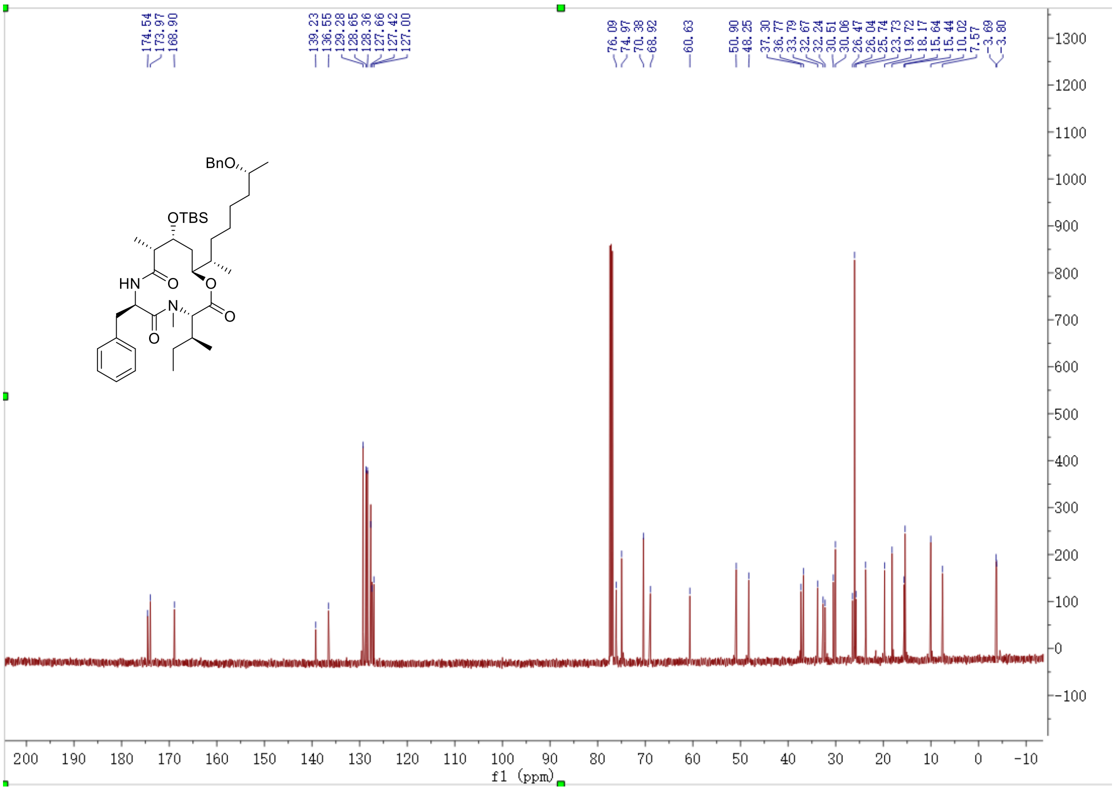

Figures S27 ^{1}H NMR Spectrum of **4**

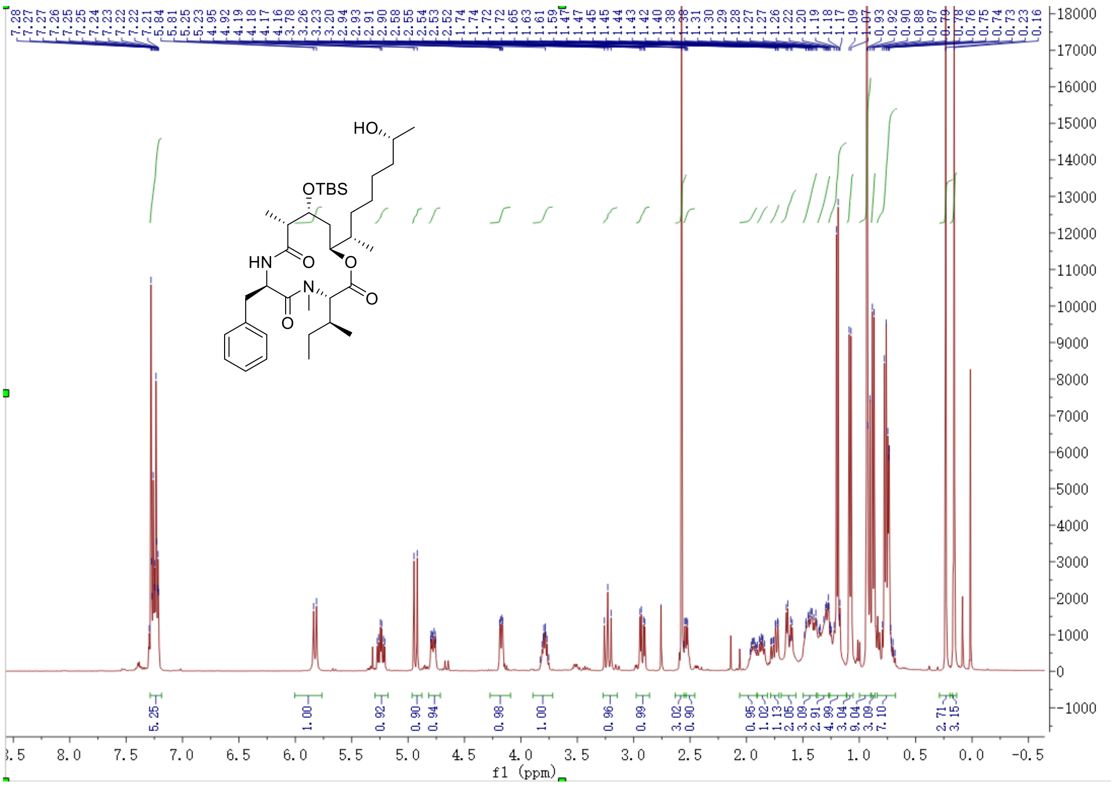

Figures S28 ^{13}C NMR Spectrum of **4**

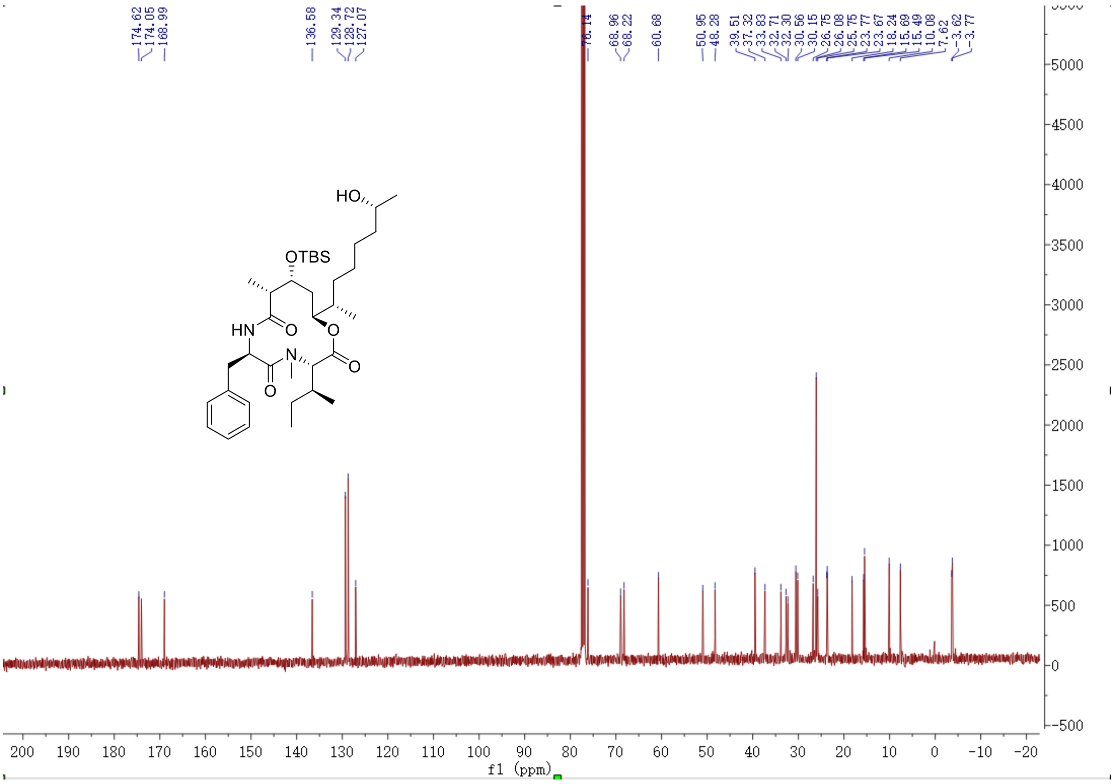

Figures S29 ^{1}H NMR Spectrum of **25**

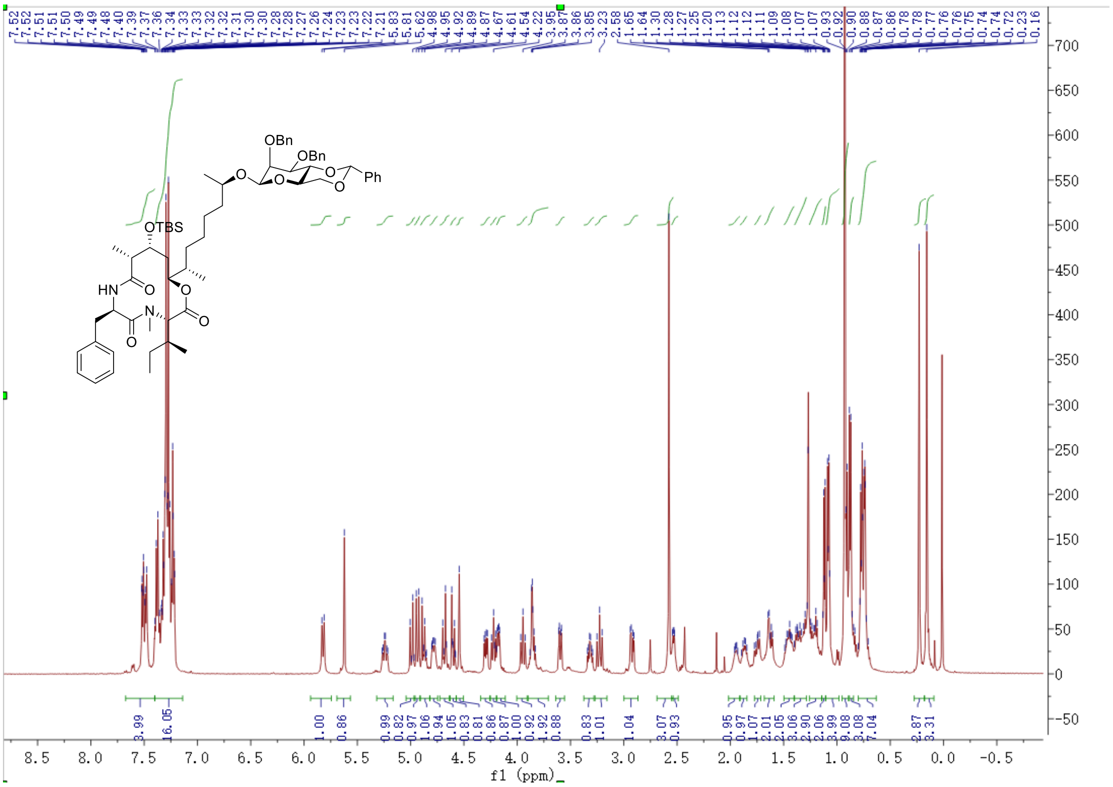

Figures S30 ^{13}C NMR Spectrum of **25**

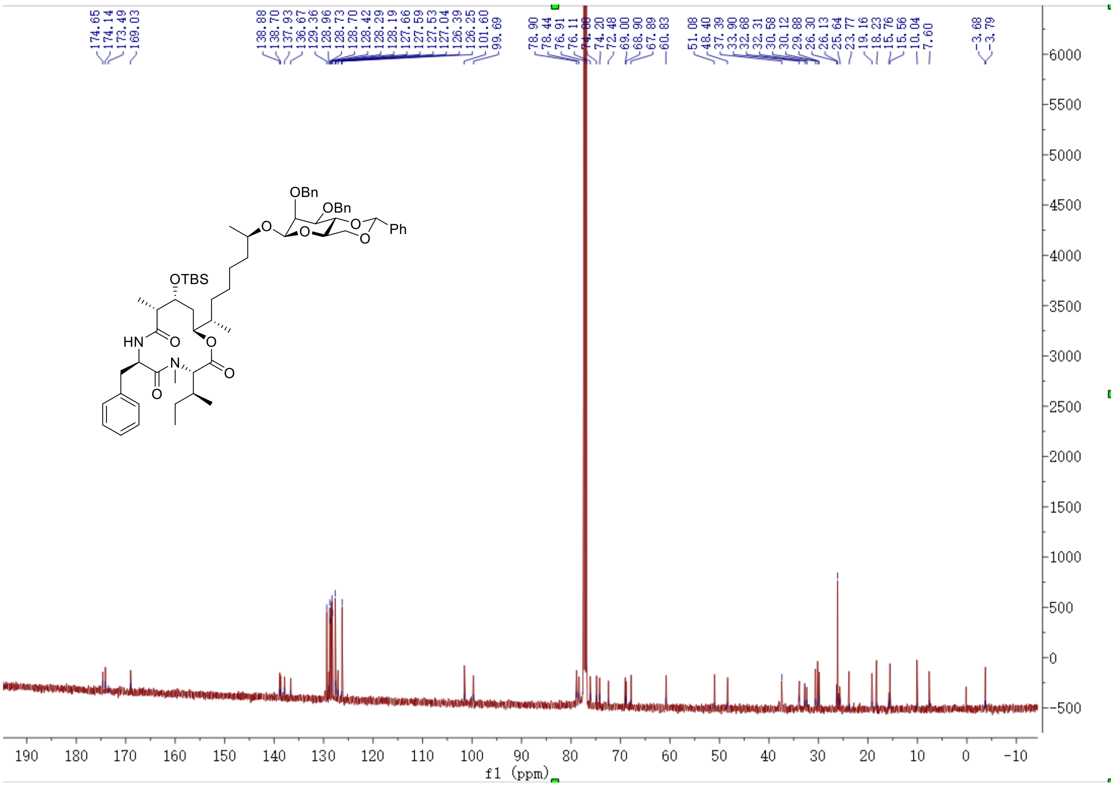

Figures S31 ^{1}H NMR Spectrum of **26**

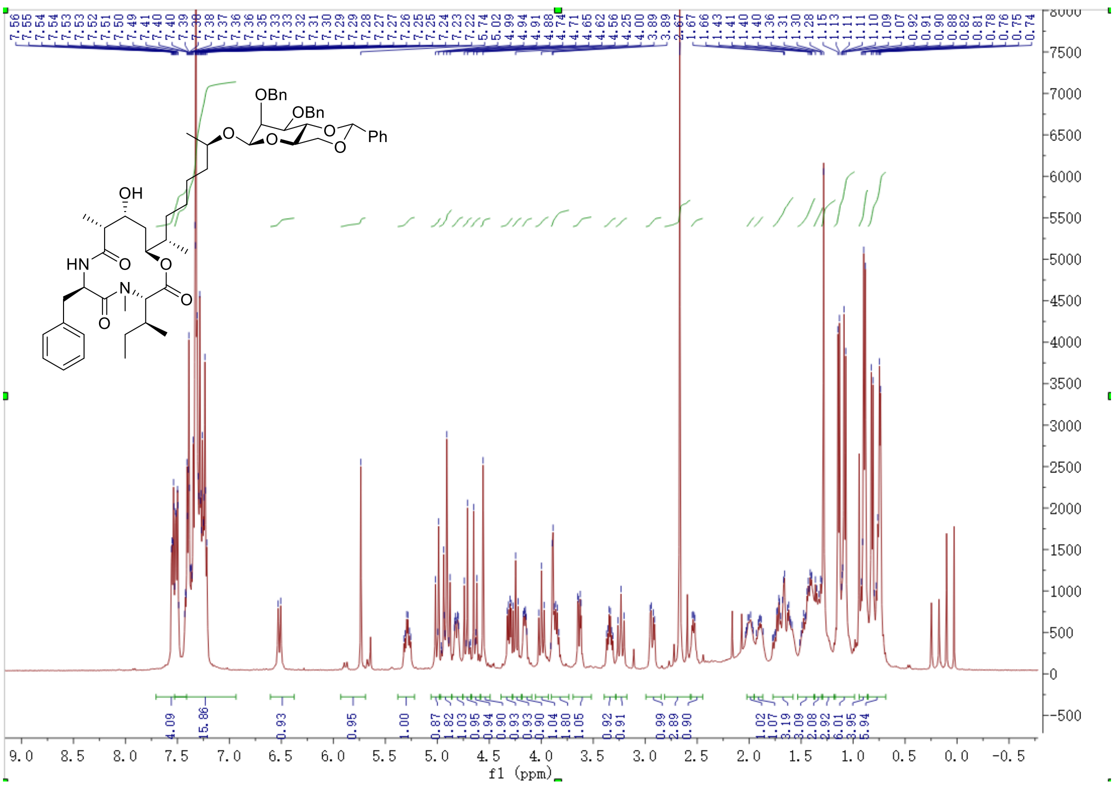

Figures S32 ^{13}C NMR Spectrum of **26**

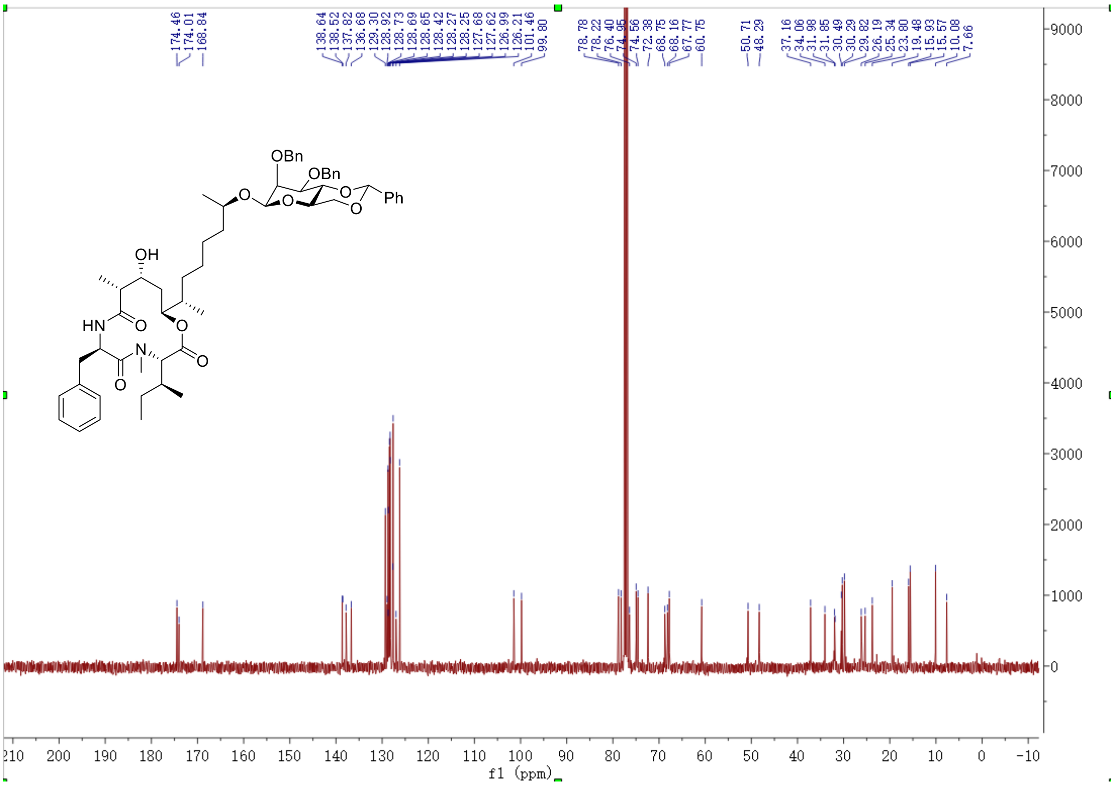

Figures S33 ^{1}H NMR Spectrum of **colletotrichamide A**

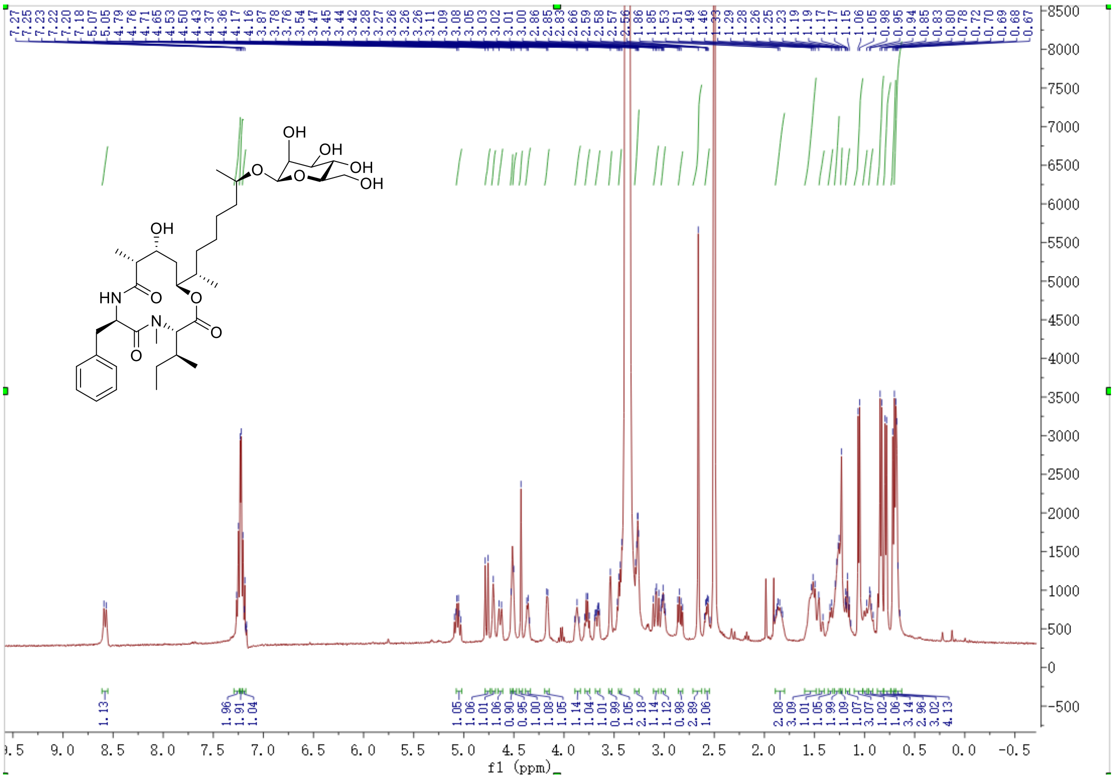

Figures S34 ^{13}C NMR Spectrum of **colletotrichamide A**

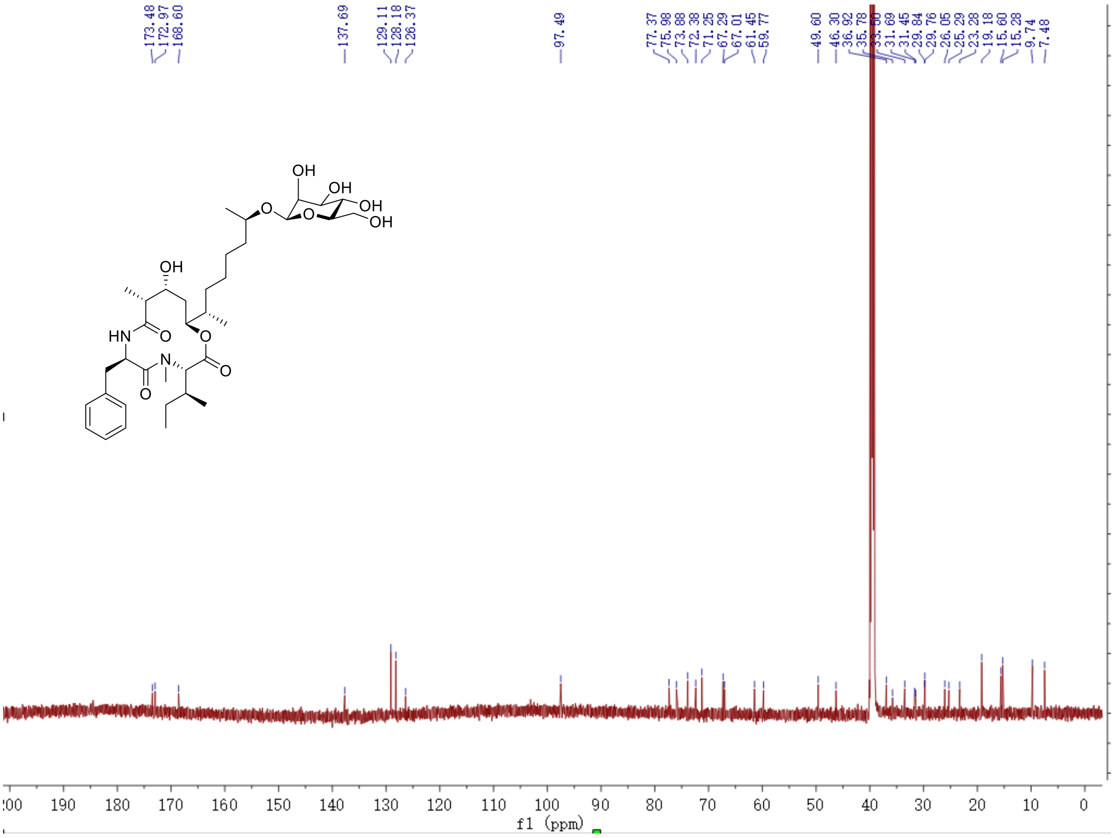

Figures S35 ^{1}H NMR Spectrum of **colletopepetide A**

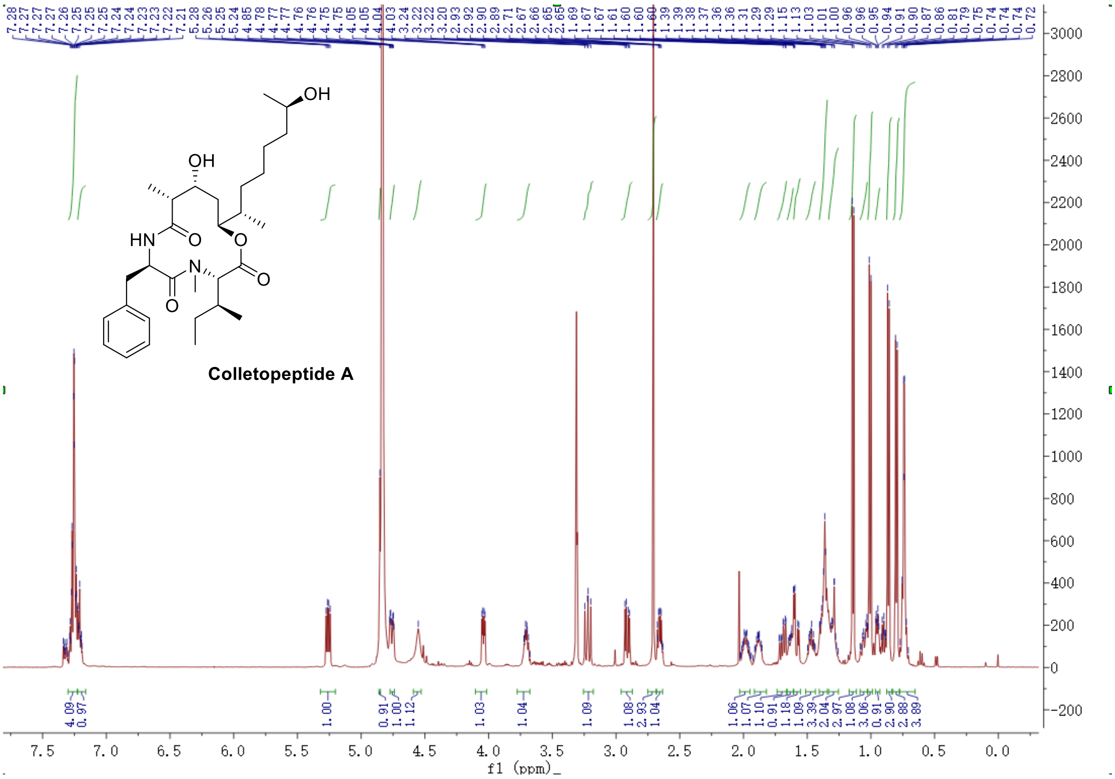

Figures S36 ^{13}C NMR Spectrum of **colletopepetide A**

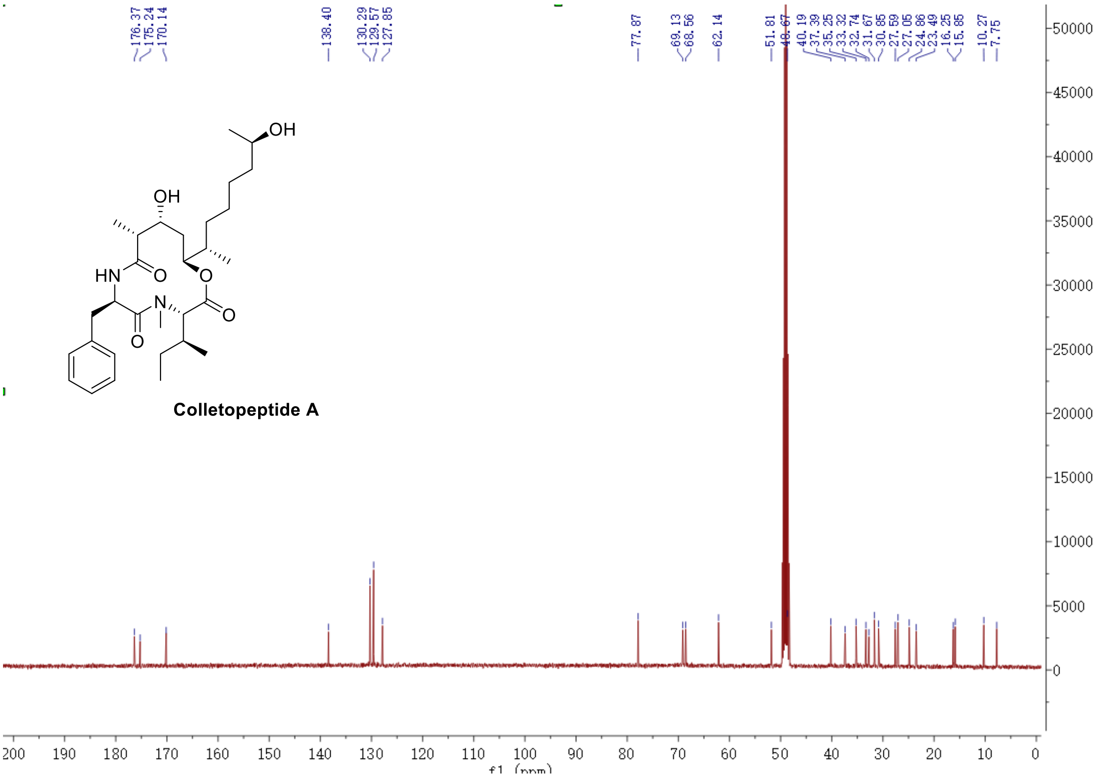

Supplement: Supplementary file 1 [file molecules-28-07194-s001.zip › molecules-2613631-supplementary.pdf]
